# PSnpBind-ML: predicting the effect of binding site mutations on protein-ligand binding affinity

**DOI:** 10.1186/s13321-023-00701-3

**Published:** 2023-03-02

**Authors:** Ammar Ammar, Rachel Cavill, Chris Evelo, Egon Willighagen

**Affiliations:** 1 Department of Bioinformatics—BiGCaT, NUTRIM, Maastricht University, Maastricht, The Netherlands; 2 Department of Advanced Computing Sciences, Maastricht University, Maastricht, The Netherlands

**Keywords:** Binding affinity, Mutation effect, SNP, Binding site, Machine learning, Feature engineering, Predictive model, Random forest

## Abstract

Protein mutations, especially those which occur in the binding site, play an important role in inter-individual drug response and may alter binding affinity and thus impact the drug’s efficacy and side effects. Unfortunately, large-scale experimental screening of ligand-binding against protein variants is still time-consuming and expensive. Alternatively, in silico approaches can play a role in guiding those experiments. Methods ranging from computationally cheaper machine learning (ML) to the more expensive molecular dynamics have been applied to accurately predict the mutation effects. However, these effects have been mostly studied on limited and small datasets, while ideally a large dataset of binding affinity changes due to binding site mutations is needed. In this work, we used the PSnpBind database with six hundred thousand docking experiments to train a machine learning model predicting protein-ligand binding affinity for both wild-type proteins and their variants with a single-point mutation in the binding site. A numerical representation of the protein, binding site, mutation, and ligand information was encoded using 256 features, half of them were manually selected based on domain knowledge. A machine learning approach composed of two regression models is proposed, the first predicting wild-type protein-ligand binding affinity while the second predicting the mutated protein-ligand binding affinity. The best performing models reported an RMSE value within 0.5 $-$ 0.6 kcal/mol^-1^ on an independent test set with an R^2^ value of 0.87 $-$ 0.90. We report an improvement in the prediction performance compared to several reported models developed for protein-ligand binding affinity prediction. The obtained models can be used as a complementary method in early-stage drug discovery. They can be applied to rapidly obtain a better overview of the ligand binding affinity changes across protein variants carried by people in the population and narrow down the search space where more time-demanding methods can be used to identify potential leads that achieve a better affinity for all protein variants.

**Supplementary Information:**

The online version contains supplementary material available at 10.1186/s13321-023-00701-3.

## Introduction

Approved drugs on the market show more side effects and variable efficacy in clinical practice than in the randomized control trials (RCT) that form the basis of their approval. RCT conducts trials on highly selective groups of people, and it is performed under tightly controlled settings. RCTs follow the assumption that the research results of the selected sample of people resemble the entire sampled population [1]. Moreover, studies showed that marketed drugs have not been as effective as expected for 40–70% of patients, with clinical practice showing them to have insufficient efficacy [2]. Furthermore, drugs that have a low therapeutic index TI (the lethal dose in 50% ($LD_{50}$) of animal population over the effective dose in 50% ($ED_{50}$) of animal population) and a narrow margin of safety (TI value close to one) tend to result in a higher number of patients developing severe adverse side effects and experiencing toxicity [3]. The observation of large variability in drug response among patients and susceptibility to side effects requires a shift towards precision medicine [4].

Therefore, over the last 50 years, pharmacogenomics has studied the genetic basis for inter-individual drug response variability [5]. Many factors are involved in patient-drug response including environmental and behavioral factors. At the same time, genetic factors also play an essential role [3]. Genetic factors that can have functionally substantial consequences on drug response are numerous. For example, they include the genetic variants’ effects on protein structure and stability, DNA transcription, and mRNA regulation [5]. Studies have shown that 80% of patients carry at least one functional variant in the drug targets of the top 100 commonly prescribed drugs in the United States [6].

Many studies [7–13] have shown relations between single nucleotide polymorphisms SNPs and drug response and toxicity. For example, a 2019 study identified novel SNPs associated with severe toxicity of 5-FU, a common chemotherapeutic agent. A change in the binding of DPYD repressor to the SNP rs72728443 suggests a mechanism by which liver DPYD, a detoxifying enzyme that metabolizes 5-FU, expression is decreased [7]. Another study showed that eight positions in the CCR5 receptor have SNPs that suggest altered responses in patients treated for HIV infection [8]. In the same study, ligand binding affinity to 24 GPCR receptors with 49 experimentally tested mutations was assessed. A five-fold change in the affinity or potency was shown to at least one of the tested ligands. The Manish et al. study in 2019 showed that Cytochrome P450 2C9 includes 6 SNPs associated with the variable enzyme activity of tamoxifen [9]. The review of Oliveira-Paula et al. mentioned that the common SNP rs1801253 (Arg389Gly) contained in the beta1-adrenergic receptor ADRB1, the main target for all beta-blockers, resulted in better blood pressure response to metoprolol for patients carrying the Arg allele [10]. Bessman et al. showed that epidermal growth factor (EGF) protein receptor expressed an increase in ligand binding affinity due to mutations in glioblastoma [11]. Toy et al. suggested that ligand-binding domain mutations in the estrogen receptor (ESR1) mediate clinical resistance to hormonal therapy in breast cancer [12]. Lastly, Fanning et al. also demonstrated that somatic mutations in ESR1 lead to anti-estrogen endocrine therapy resistance [13].

The variation in drug-response at the protein level and its underlying mechanisms are of significant interest in developing new drugs with an estimate of six SNPs affecting five different FDA-approved drugs carried by every individual [14]. Hence, being able to predict the effect of mutations on drug-protein interactions has a notable benefit in drug discovery. SNPs may occur anywhere in the protein and not all of them lead to mutations on the amino acid level since the 20 proteinogenic amino acids can be encoded by 64 nucleotide triplets or codons. Moreover, even when an amino acid is mutated into another one, the location of the mutation, its type and the role that amino acid plays in the protein structure and function largely affects the impact of such a mutation. Hence, the changes in the protein resulting from a single amino acid substitution maybe too small to be reflected on the protein level, and for drug-binding, those mutations that occur in the binding site are the most likely to influence the binding affinity.

Having such a model will save time and costs for virtual screening and, at the same time, give the ability to screen the ligand against the target protein with all known binding site variants. Hence, it will report more realistic binding affinity, capture a wide range of populations’ genetic makeup, and help develop drugs that show more consistent efficiency across different populations. Being able to predict mutation effects would also help in the area of precision medicine where drugs and doses can be chosen following the genetic makeup of the patient to avoid adverse side effects and maximize the drug response.

## Related work

The problem of predicting mutation’s effect on protein structure and function is well studied in the literature with a scope ranging from predicting mutation effect on protein sequence [15–17] to 3D mapping of mutations onto protein structures with visualization and highlighting their impact [18, 19]. Many studies focused on SNP-related problems like their effect on protein-protein binding interactions [20], transcription factor binding [21–23], cell signaling [24, 25] and protein stability [26–29]. However, the specific impact of missense mutations in the binding site on protein-ligand binding affinity is much less covered. Also, studies often focus on a small set of mutations in a specific target protein. In the following paragraph, we highlight some of the related works that studied protein-ligand binding affinity or mutations’ effect on it.

 Choudhury et al. used a data mining approach to integrate multiple resources to identify single-nucleotide variants (SNV) that occur at drug-binding sites and their effects. They used data sources including datasets for genetic and clinical variations, chemical structures, drugs, protein-ligand structure complexes, and drug targets [30]. Schneider et al. proposed a machine learning random forest regression model to predict the protein-ligand binding affinity using structure and ligand descriptors. The model achieved a correlation coefficient of 0.73 on the internal test set [31]. However, this study did not incorporate mutation effects on the binding affinity, but their approach is relevant to our research goals. Shaikh et al. used a proteochemometric modeling approach to predict drug-target interactions using machine learning models. The authors formulated the problem as a classification problem where positive instances are protein-ligand complexes with ligand’s activity value equal to or larger than 1 $\mu$M against the protein target. The features used to train the models included sequence and structure-based descriptors for proteins, structural descriptors for the binding pocket as implemented in FuzCav fingerprint, and Morgan circular fingerprint as ligand descriptors with the highest AUC being 89% [32]. Pires et al. used a Gaussian processes machine learning approach to predict protein-ligand binding affinity (the method called CSM-lig). They used cutoff scanning matrix (CSM), a graph-based signature, to represent the 3D structural environment of proteins and ligands [33]. The same authors presented in a different study a regression model to predict the effect of single-point missense mutations on ligand affinity. The method named mCSM-lig used graph-based features to encode geometrical and physicochemical properties for the proteins and the protein-ligand complex. The regression model achieved a Pearson correlation coefficient of 0.627 over the entire dataset and 0.737 after 10% outlier removal [34]. A third study for the same authors resulted in mCSM-AB, a method to predict the antibody-antigen affinity changes upon mutation in terms of Gibbs Free Energy with limited applications for antibody engineering and development. The method used graph-based structural signatures to train a machine learning regression model and achieved a Pearson correlation coefficient of 0.53 on 10-fold cross-validation [35]. Kim et al. built the mutLBSgeneDB database using an integrative multi-source approach. The database included genetic, protein structure, ligand-binding site mutations, differential gene expression, gene-gene network, and phenotype information from several sources integrated into this database. The mutLBSgeneDB database also contained drug binding affinities for drugs and their targets selected as the top 20 ranked genes [36]. Petukh et al. proposed a new methodology termed Single Amino Acid Mutation based change in Binding Free Energy (SAAMBE) to predict the changes of the binding free energy upon mutations in protein-protein complexes. The method predicted the binding free energy change upon single-point mutations achieving a Pearson correlation coefficient of 0.62 [37]. Sawada et al. presented a benchmarking study for a wide range of chemical descriptors for drug-target interaction prediction. The authors formulated the problem as a classification problem (interact, does not interact). The study compared 18 chemical descriptors of drugs (e.g., CDK, KlekotaRoth, MACCS, ECFP, KlekotaRoth, FCFP, E-state, MACCS, PubChem, graph kernels, Dragon, and KCF-S) and four descriptors of proteins (e.g., domain profile, local sequence similarity, amino acid composition, and string kernel) on  100,000 drug-target interactions. The KCF-S descriptor resulted in the best prediction accuracy [38]. KCF-S (KEGG Chemical Function and Substructures) uses the information of chemical structure conversion in enzyme reactions to encode different levels of substructures and functional groups. KCF-S descriptor is composed of seven attributes: atom, bond, triplet, vicinity, ring, skeleton, and inorganic. These can be used for many applications like structure-based molecule clustering and machine learning [39].

None of the previous studies were performed on a large-scale dataset of proteins and mutations, and specifically on the binding site mutations to predict their effect on binding affinity. Hence, our research aims to fill this gap and build a machine learning model trained on a relatively large dataset of protein variants with an application focus on drug discovery and precision medicine.

## Methods

**Fig. 1:**
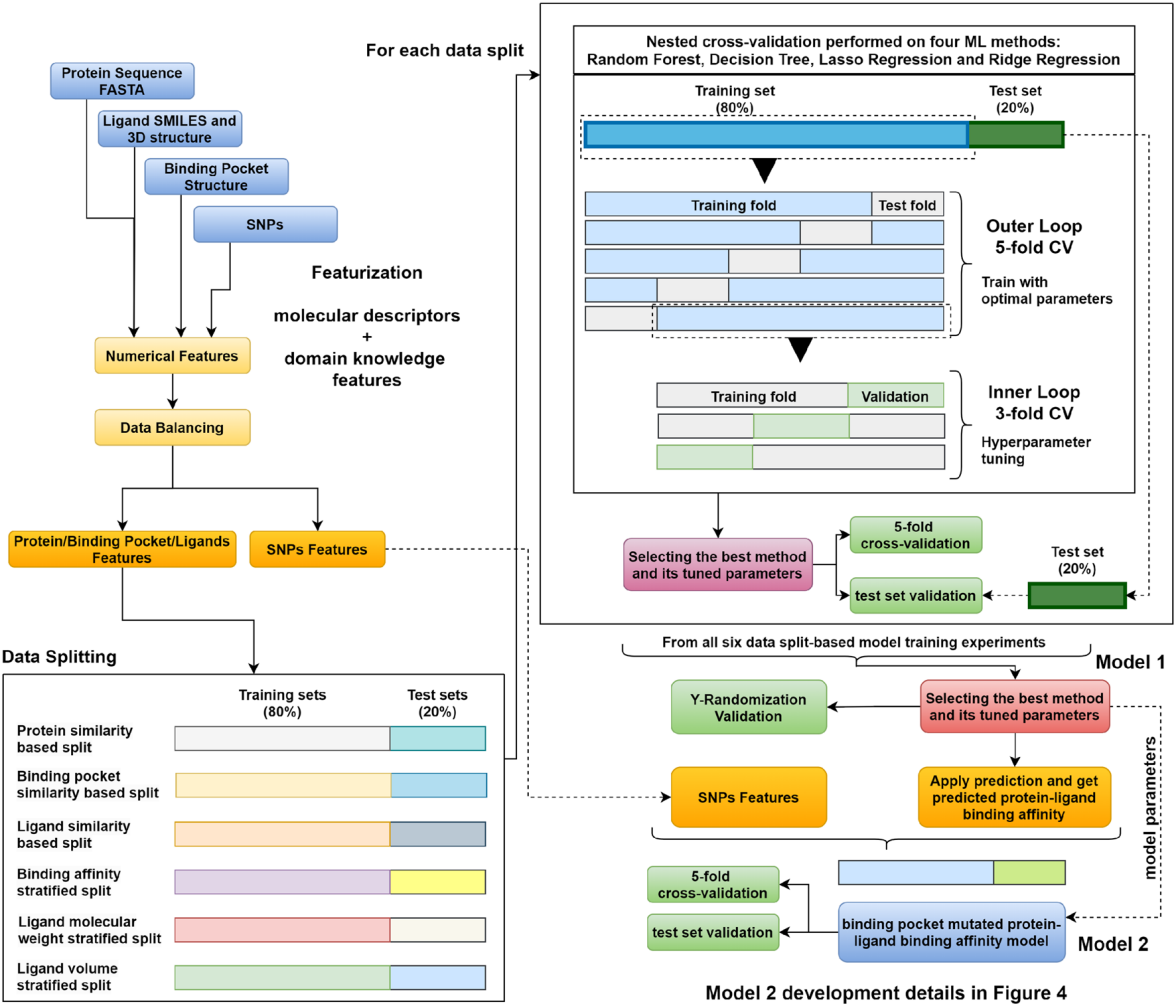
Overview of PSnpBind-ML methodology composed of two regression models. The first model predicts wild-type protein-ligand binding affinity using the numerical representation of the protein, ligand and the binding site. The second model uses the wild-type protein-ligand binding affinity besides numerical representation of the mutation to predict the mutated protein-ligand binding affinity

The methodology of this work aims at building a machine learning model that predicts the protein-ligand binding affinity of wild-type proteins and their variants with single-point binding site mutations. Figure 1 shows the proposed approach which is composed of two regression models. The first model predicts wild-type protein-ligand binding affinity using the numerical representation of the protein, ligand and the binding site. The second model uses the wild-type protein-ligand binding affinity besides numerical representation of the mutation to predict the mutated protein-ligand binding affinity.

### Data sources

PSnpBind [40] is the main data source used in this work to obtain information about proteins, binding pocket mutations and ligands that are docked to both the wild-type protein and its variants. PSnpBind is a large database of protein-ligand complexes covering a wide range of binding pocket mutations and a large small molecules’ landscape. It is primarily designed for applications like developing machine learning algorithms to predict protein-ligand affinity or single-point mutation effect on it. The PSnpBind database contains around 600,000 protein-ligand complexes covering 730 protein variants for 26 proteins and more than 32,000 ligands. Moreover, PDBbind [41] was used as a resource for the annotated binding pockets of the proteins in order to generate numerical features from them. Since PSnpBind is constructed using the structures from PDBbind, it was straightforward to use the same structures for feature engineering. The core set of PDBbind 2016 (also known as CASF 2016) was used in this work.

### Feature engineering

The following sections describe the groups of descriptors/features used to build the dataset for machine learning training and testing.

#### Protein features

Ain et.al [42] study conducted a benchmark of 21 protein descriptors used in modeling ligand selectivity. The Sequence-Order-Coupling Number (SOCN), along with amino acid and dipeptide composition showed a better performance than other sequence-based features like quasi sequence order (QSO) and composition, transition and distribution (CTD), and ProFeat descriptors. SOCN is a protein descriptor composed of 60 values. It reflects the indirect effect of the protein sequence order by calculating the coupling factor according to the physicochemical distance between coupled residues based on the Schneider-Wrede distance matrix [43]. Schneider-Wrede distance matrix is derived from hydrophobicity, hydrophilicity, polarity, and side-chain volume properties of amino acids. Protr R package (v1.6-2) [44] was used to generate the protein features. Protr is a freely available and open-source R package that calculates various commonly used structural and physicochemical descriptors from protein sequences and properties retrieved from the AAindex database [45].

#### Binding pocket features

Four main aspects were considered when designing features for the binding pocket: secondary structure, buried and exposed residues, accessible surface area, and binding pocket volume. BioJava library (v5.3.0) [46] was used to compute the features of the binding pockets.

- Secondary structure: the percentages of residues involved in the binding pocket belonging to each of the secondary structures (Helix, Strand, Other) were used as features. Further, the dominant secondary structure that pocket residues are composing was also used as a feature.
- Accessible surface area: the total accessible surface area (ASA) of the binding pocket was used as a feature. Total ASA was calculated as the sum of the ASA of all residues annotated as part of the binding pocket.
- Buried and exposed residues: Each residue with a relative ASA of 20% or less was considered buried. In contrast, Each residue with a relative ASA of more than 20% was considered exposed. The number of buried residues, the number of exposed residues, and the ratio of the number of buried to exposed residues were used as features.
- Binding pocket volume: the volume was calculated using ProteinVolume v1.3, a tool to compute the geometric volume of proteins, in this case, the binding pocket structure [47].

#### Mutation features

Amino acid mutation features were designed to capture both physicochemical and structural changes in the mutated residue’s local environment. BioJava library was used to compute the features of mutations. Following is the description of the designed features:

- Secondary structure information: protein secondary structure (SS) is the smallest three-dimensional structure formed from the polypeptide chain upon folding. The DSSP algorithm was used to obtain the 8-class SS features [48]. Another simplified version of the secondary structure was included where the mutation residue was assigned to one of three groups of SS (Helix, Beta Strand, or Other).
- Specific amino acid mutations: cysteine, glycine, and proline are three amino acids that play unique roles in protein structure. Cysteine forms disulfide bridges with another cysteine residue, an essential component of the secondary and tertiary structures. Cysteine also binds to Zn metal ions in the binding pocket resulting in an important complex for the protein structure [49]. The backbone flexibility is substantially affected by the conformational flexibility of glycine side chains and the rigidity of proline side chains [50]. Large structural effects can take place by mutations from or to one of these three amino acids. Three binary terms were included as features to capture if the mutation was from or to glycine, proline, or cysteine.
- Amino acid group changes: the twenty amino acids were grouped into three groups for each of the seven types of physicochemical properties: hydrophobicity, normalized Van der Waals volume, secondary structures and solvent accessibility, polarity, polarizability, and charge [51]. Each mutation has nine possibilities of being changed from one group to another for each attribute (3x3 possibilities). The features were encoded as categorical variables with nine possible values.
- Mutation residue amino acid and surrounding properties: A combination of 48 amino acid properties analyzed in a previous study for relations to protein stability [52] were used to calculate two sets of features. First, for each property, a change induced by the mutation is calculated using a simple formula [P(i) = P_mutation_(i) − P_WT_(i)] [53]. Second, the influence of the local structural environment surrounding the mutation residue was incorporated for each of the AA properties. The amino acids were represented by their alpha carbon atoms, and the surrounding residues (j) within the sphere of radius 8 Å [54] were selected. The surrounding features for each property were calculated using the formula: P_surr_(i) = [$\sum _{j} P_{j}$] − P_mutation_(i).
- Solvent Accessible Area (ASA) change: Solvent accessibility of a residue was calculated with BioJava using the rolling ball algorithm [55], and the relative ASA was obtained for both wild-type and mutated residues. Relative ASA is the ASA of the residue with respect to its ASA in an extended tri-peptide conformation (GLY-x-GLY) [56]. The ratio between relative ASA values (ASA_mutation_ − ASA_WT_) was used as a feature.
- Phi and Psi dihedral angles: In chemistry, a dihedral angle is an angle between planes defined by two sets of three atoms, having two atoms in common. These angles have restrictions for their values, reflecting energetically allowed regions for backbone dihedral angles. So, changes in dihedral angles upon mutation capture structural changes in the backbone of the protein chain.
- FoldX energy terms: FoldX [57], a software used to introduce mutations to the protein structure in PSnpBind, produces 22 energy-term changes between the wild-type and the mutated versions of the protein. Those terms obtained during the construction of the PSnpBind database were used to describe the structural effect of the mutation on the protein [40].

#### Ligand features

Chemistry Development Kit v2.3 [58, 59] was used for molecule standardization and molecular descriptors generation. Ligands, obtained as SMILES strings, were parsed using the CDK and several steps were applied to normalize them. First, atom type perception and atom configuration normalization was applied. Next, implicit hydrogens were removed and re-added using the CDKHydrogenAdder function and then converted to explicit hydrogens. Finally, aromatic system identification and kekulization were applied to all molecules. Chemical descriptors covering four layers of chemical representation (0D, 1D, 2D and 3D) were calculated and used to represent the ligands as numerical vectors. The code was implemented using Java, an object-oriented programming language.

### Data balancing

The PSnpBind database contains a large imbalance in the number of ligands docked to each protein (ranging from 119 to 7058). Since ligands capture structural and functional information for the proteins they bind to, that could make the ML model biased toward learning the representation of the proteins (and their pockets) with the highest number of ligands. It would also learn to recognize ligands that fit certain proteins more than the others since their number is relatively large in the dataset. For the previous reasons, the data composition was balanced before splitting by selecting 350 ligands for each protein. Two proteins had less than 350 ligands binding pairs, and for these two cases, all ligands were considered. The ligands were sampled with respect to the Tanimoto index distribution since the rationale behind using a low value for it was to include a wide range of ligands that would show different binding affinities.

### Dataset preparation

The features described in the previous sections were used in conjunction with the docking binding affinity results from PSnpBind and different splits were applied to create the datasets to be used in model training and validation. For preprocessing, the features that had zero values or near zero variance were removed. Next, all instances with extreme binding affinity values falling outside the range [− 16, − 4] kcal/mol were excluded. Finally, all categorical values were encoded as binary arrays of features using the One Hot Encoding technique in sklearn v0.24.0 Python package [60].

### Data splitting

Data splitting was carried out to explore its effect on the machine learning models performance, incorporating three levels of similarities among the dataset instances (protein, binding site, and ligand) besides the random splitting stratified on three variables (binding affinity, ligand molecular weight and volume). Different train-test datasets were generated on which machine learning models were trained and validated.

#### Split 1: Protein similarity-based splitting

Proteins with similar sequences tend to have similar structures [61–63]. Therefore, having proteins in the training set that are similar to the ones in the test set can cause leakage issues to the model. Hence, splitting the dataset by protein similarity could help examining such a case if any. Protein sequence clustering was performed in order to split the data on the bases of protein similarity, where sequence clustering algorithms try to group protein sequences that share similarities in clusters [64]. UCLUST [65], is a sequence clustering algorithm included in the USEARCH sequence analysis tool, was used for this purpose. Sequence identities are computed using a global alignment method, and every sequence, in order to be added to the cluster, should have a similarity with the centroid above a defined threshold. UCLUST is reliable at identity scores of  50% and above for proteins and  75% and above for nucleotides. The effectiveness of this method becomes questionable at low identity scores because of the degrading alignment quality. Besides, homology cannot be reliably determined from the alignment.

UCLUST pre-compiled 32-bit executable for Linux was used, downloaded from https://drive5.com/usearch. Sequences for the 26 proteins in PSnpBind were obtained from UniProt using their UniProt IDs. Then, sequences were ordered by sequence length using the “-sort” parameter. For further investigation, after clustering did not work as expected (see the results section), an all-against-all protein-protein sequence similarity search was performed using “BLASTp”, a tool from the NCBI BLAST tool suite [66]. An E-value cutoff of 10^-5^ was used using the “-evalue” parameter.

Another approach was explored, protein family domains annotation using the Pfam database. A local version of the Pfam [67] database (version 32.0) was downloaded. Then, the Pfam database was prepared for use with HMMER v3.3 [68] (“hmmpress” tool), which is the same program that Pfam site uses to search for protein domains in submitted queries. The “hmmsearch” command was used to perform a search against the Pfam database using the 26 protein sequence and an E-value cutoff of 10^-5^.

#### Split 2: Binding site similarity-based splitting

Proteins that share no distinct global (sequence or structural) similarity can still share similar binding sites [69]. Hence, they can bind to similar ligands. Therefore, splitting the dataset by binding site similarity can help observe how that affects the model performance. FuzCav [70] was used to calculate the all-against-all similarity between the binding pockets. Ehrt et al. published in 2018 an exhaustive evaluation study to benchmark binding sites comparison methodologies [71]. The study grouped these methods into three groups (residue-based, surface-based, and interaction-based), where FuzCav belongs to the residue-based group. FuzCav was the only method that fulfilled all four aspects of quality covered in the study (site definition, similarity ranking, completeness, and run time). FuzCav featurizes druggable protein-ligand binding sites using a 4833 long integer vector. It can also be applied to any protein and binding cavity. SimCalc tool in the FuzCav package was used to calculate pairwise similarity for all the binding pockets (Additional file 1: Table S3). The authors of FuzCav showed in their research that a similarity threshold of 0.16 could be used to identify similar binding sites and the same threshold was used to filter the pairwise similarity results in this work.

#### Split 3: Ligand similarity-based splitting

Ligands that bind to proteins can share structural and functional similarities. Chemical characteristics of ligands are also known to capture the functional and mechanistic properties of proteins. Therefore, splitting the dataset by ligand similarity can help detect if the ligand similarity has an effect on the model’s performance and the potential data leaking problems resulting from it. RDKit Python library v2022.3.4 was used to select 20% of the most dissimilar ligands to the remaining 80% using the sphere exclusion algorithm. Next, all instances related to the 20% ligands were used as a test set and the remaining 80% of instances formed the training set.

#### Split 4–6: Stratified random splitting

In this scenario, splitting with stratification on three variables (the binding affinity, ligand’s molecular weight and ligand’s volume) was performed resulting in three different data splits each having 80% train set and 20% test set. The caret R package [72] was used for this purpose. The function “createDataPartition” provided by “caret” creates balanced splits of the data based on a selected variable preserving its overall distribution.

### Chemical space characterization

The chemical space for all the obtained train-test splits was characterized by the scattered distributions of the first two principal components derived from the principal component analysis (PCA) for 53 out of 54 molecular descriptors (the feature “rule of five” violations was excluded because it is not numeric) and by the scattered distributions of molecule weight and atom-additive octanol-water partition coefficient (XlogP) [73].

### Machine learning models training and validation

The problem under investigation was formulated as a regression problem to predict the protein-ligand binding affinity taking into consideration single-point mutation information and its effect on the binding affinity. This task was accomplished over two steps: model training, and evaluation.

#### Machine learning modeling

Four machine learning methods were compared for their ability to predict protein-ligand binding affinity (model 1), namely, random forest, decision tree, lasso regression and ridge regression.Training multiple methods helps estimating the influence of selecting the modeling method. For the linear regression models, standard scaling was applied on the datasets (independent variables) before training. Nested cross-validation (CV) was carried out to select the best model and perform parameter tuning [74]. For the outer loop, 5-fold CV was used and 3-fold CV for the inner loop as depicted in Fig. 1. Without nested cross-validation, model selection uses the same data to evaluate model performance and fine-tune model parameters, which could result in an optimistically biased evaluation of the model. The model resulted from the best performing ML method and its tuned parameters for each data split was further validated using an independent test set and the best model parameters among all data split based models was used to train the mutated protein-ligand binding affinity model (model 2). The implementation of sklearn v0.24.0 Python package was used for the chosen model types. Finally, feature importance was obtained from the models and the relevant plots were provided.

#### Model evaluation

Three approaches for model validation were followed. First, nested cross-validation was applied to all models. Next, to ensure our models were independently evaluated, the best model resulting from each data split was validated against an independent test set. Further, a third validation approach was applied which is Y-randomization, or response permutation testing. It is an approach to estimate the risk of chance correlations [75]. Y-randomization was applied by keeping the features space fixed and randomly shuffling the binding affinity (Y variable) and then retraining the model. The process was repeated ten times, each time with a different randomized dependent variable vector. The performance of each model was evaluated using four metrics, coefficient of determination (R^2^), Root Mean Square Error (RMSE), Mean Absolute Error (MAE), and Mean Squared Error (MSE). Moreover, two metrics were provided to compare the error of the obtained models against a dummy regression model predicting the mean of the dependent variable and which serves as a base model. The two metrics are relative absolute error (RAE) and root relative squared error (RRSE) which are also used in other machine learning packages like WEKA [76]. The previously mentioned metrics were calculated as follows:

1 $$\begin{aligned} R^2 = 1 - \frac{ \sum _{i=1}^{n_{test}}(Y_{obs}-Y_{pred})^2}{ \sum _{i=1}^{n_{test}}(Y_{obs}-\bar{Y}_{train})^2} \end{aligned}$$

2 $$\begin{aligned} MAE = \frac{ \sum _{i=1}^{n_{test}} |Y_{obs}-Y_{pred} |}{n_{test}} \end{aligned}$$

3 $$\begin{aligned} MSE = \frac{ \sum _{i=1}^{n_{test}}(Y_{obs}-Y_{pred})^2}{n_{test}} \end{aligned}$$

4 $$\begin{aligned} RMSE = \sqrt{ \frac{ \sum _{i=1}^{n_{test}}(Y_{obs}-Y_{pred})^2}{n_{test}}} \end{aligned}$$

5 $$\begin{aligned} RAE = \frac{MAE_{RF\ model}}{MAE_{dummy\ model}} \end{aligned}$$

6 $$\begin{aligned} RRSE = \frac{RMSE_{RF\ model}}{RMSE_{dummy\ model}} \end{aligned}$$

## Results and discussion

### Feature engineering

The feature engineering phase resulted in 256 features covering four layers of information: proteins (60 features), binding sites (9 features), ligands (54 features) and mutations (133 features). Table 1 shows a summary of the features and their counts for each of the four aspects of data.

Meaningful representation of proteins plays an essential role in the performance of many bioinformatics methods such as predicting protein functions [77], protein family classification [78], and predicting the interactions between protein-protein [79] and protein-ligand pairs [80]. Sequence-derived features are a common type of features that are used to represent proteins. Proteins that share the same family or domains can bind to similar ligands. The biophysical and functional properties of proteins are known to be captured by the chemical properties of their ligands. Hence, ligand-based features can be used to represent proteins [81]. Also, proteins with no global similarity can still have similar binding sites and hence bind similar ligands [69]. Therefore, features representing the binding site of the target proteins were included in order to capture those similarities when proteins themselves are not similar. Lastly, since the aim of this work is to predict the binding affinity for proteins with different single-point mutations in their binding site, mutations here introduce another level of complexity that needs to be explicitly encoded using unique features enabling the machine learning model to capture their effect on the binding affinity. For example, the mutation’s amino acid group change and the physicochemical properties of the mutation’s surrounding residues.

**Table 1:** Features and descriptors breakdown for protein, binding pocker, mutation and ligand representation

Protein, binding site and mutation features (202 features)	
Protein (60 features)	Sequence-Order-Coupling Number (SOCN) descriptor
Binding site (9 features)	Secondary structure (4 features) Accessible surface area (1 feature) Buried and exposed residues (3 features) Binding pocket volume (1 feature)
Mutation (133 features)	Secondary structure information (2 features) Cysteine, glycine, and proline mutations (3 features) Amino acid groups changes (7 features) Properties of mutated AA and its surrounding (96 features) Solvent Accessible Area (ASA) change (1 feature) Phi and Psi dihedral angles (2 features) FoldX energy terms (22 features)

Ligand features (54 features)	
Descriptor category	Descriptors/Fingerprints
0D Descriptors	Molecular weight (1 feature) Aromatic atoms count (1 feature) Aromatic bonds count (1 feature) All atoms count (1 feature) N atoms count (1 feature) O atoms count (1 feature) Electronegativity (1 feature)
1D Descriptors	Number of Hydrogen donors (1 feature) Number of Hydrogen acceptors (1 feature) Number of rotatable bonds (1 feature) Number of violations of Lipinski’s rule (1 feature) Basic groups count (1 feature) XlogP (1 feature) AlogP (3 features) JPLogP (1 feature)
2D Descriptors (Topological)	BCUT eigenvalue based descriptor (6 features) Topological polar surface area (1 feature) Fractional polar surface area (1 feature) Small rings count of sizes 3-9 atoms (9 features) Vertex adjacency information (1 feature) Carbon connectivity types (9 features) Atomic polarizabilities descriptor (1 feature)
3D Descriptors (Geometric)	Van der Waals Volume (1 feature) Solvent accessible surface area (1 feature) Momentum of inertia (6 features) Radius of gyration (1 feature)

### Data splitting

The data used to train and test machine learning models directly affects their applicability and generalizability. Unfortunately, there is no consistency in the literature on how to split the datasets. This inconsistency makes it tricky to compare models’ applicability. Random splitting of the datasets is commonly used, which also leads to variances in the output, and it is not always best for evaluating machine learning methods [82, 83]. For example, Sheridan et al. showed for QSAR modeling on assay data that time-based split (i.e. building a model on assay data available at a certain date and tests the model on data that is generated later) gives an R^2^ that is more like that of true prospective prediction than the R^2^ from random selection (too optimistic) or from leave-class-out selection (too pessimistic). In this work, six different splits were created to evaluate the models’ prediction capability on a wide range of protein-ligand complexes.

#### Protein similarity-based data splitting

As proteins with similar sequences will probably have similar structures and hence may bind to similar ligands, protein similarity-based splitting was performed to observe such an effect. The clustering process of protein sequences using UCLUST resulted in 25 clusters for the 26 proteins included in the PSnpBind dataset used as the main data source in this work. That was unexpected and unhelpful to split the dataset by protein similarity. The likely reason for these results is that the 26 proteins share low identities among each other, and that is supported by the methodology of constructing the PDBbind dataset from which those proteins were selected in order to build the PSnpBind database. Furthermore, the pairwise similarity search confirmed that these protein sequences have a low identity among each other, where 25 out of 26 proteins have an identity of less than 50%. That explains why UCLUST was not able to find a smaller number of clusters. Additional file 1: Table S1 shows BLASTp results (excluding the similarity of the proteins against themselves).

Next, in another attempt to split proteins and that is by their family domain annotation similarity, the Pfam search resulted in 125 family hits for all the proteins (Additional file 1: Table S2). Multiple families were linked to each protein sequence. After careful examination, five out of 26 proteins were selected and found not to share any protein family with the remaining 21 proteins or among each other. Hence, those protein structures and consequently, all their mutated structures and selected ligands were used as a test set (20%) while the rest were used for the training set (80%). Table 2 shows the proteins included in the train and test sets by protein similarity.

The motive behind using protein family annotation to group the proteins is that if the proteins do not share global sequence similarity, it makes more sense to look for similarities on a smaller scale like functional domains. So, if we can group proteins by similar domains they share, then we can split them into two different sets for training and testing. The Pfam database is a large collection of protein domain families. Each family is represented by multiple sequence alignments and a hidden Markov model (HMM). After examining the Pfam website, it was found that the website offers a search function through the web UI, but it has no API for automated scripts and workflows, and that also affects the reproducibility of our research. Hence, the database preparation and search were conducted locally on Pfam v32.0.

**Table 2:** List of the proteins used in PSnpBind-ML model and the selected proteins for train and test sets based on protein similarity-based (PS) and binding site similarity-based (BSS) data split

PDB ID	Gene name	UniProt ID	Protein name	PS	BSS
1owh	PALU	P00749	Urokinase plasminogen act.	Train set	Test set
2c3i	PIM1	P11309	Pimtide protein kinase PIM1	Train set	Train set
2hb1	PTPN1	P18031	Tyrosine phosphatase type 1	Test set	Train set
2pog	ESR1	P03372	Estrogen receptor	Train set	train set
2weg	CA2	P00918	Carbonic anhydrase 2	Test set	Train set
2y5h	F10	P00742	Factor XA	Train set	Train set
3b27	HSP90AA	P07900	Heat shock protein 90-alpha	Test set	Train set
3b5r	AR	P10275	Androgen receptor	Train set	Test set
3fv1	GRIK1	P39086	Glutamate receptor	Train set	Train set
3jvr	CHK1	O14757	protein kinase Chk1	Train set	Train set
3pxf	CDK2	P24941	Cell division protein kinase 2	Train set	Train set
3u9q	PPARG	P37231	PPAR gamma	Train set	Train set
3udh	BACE1	P56817	Beta-secretase 1	Test set	Train set
3up2	AURKA	O14965	Aurora kinase A	Train set	Train set
3utu	F2	P00734	Thrombin	Train set	Train set
4crc	F11	P03951	Coagulation factor XI	Train set	Train set
4dli	MAPK14	Q16539	Human p38 MAP kinase	Train set	Train set
4e5w	JAK1	P23458	Tyrosine-protein kinase JAK1	Train set	Train set
4gr0	HME	P39900	Macrophage metalloelastase	Train set	Train set
4j21	TNKS2	Q9H2K2	Tankyrase-2	Train set	Test set
4jia	JAK2	O60674	Tyrosine-protein kinase JAK2	train set	Train set
4m0y	ITK	Q08881	Tyrosine-protein kinase	Train set	Train set
4twp	ABL1	P00519	abl1 kinase	Train set	Train set
4wiv	BRD4	O60885	First bromodomain of Brd4	Test set	Train set
5a7b	TP53	P04637	Cellular tumor antigen P53	Train set	Test set
5c28	PDE10A	Q9Y233	Phosphodiesterase 10	Train set	Test set

#### Binding site similarity-based data splitting

Since unrelated proteins may have similar binding sites that are capable of recognizing chemically similar ligands, a binding site similarity-based split was carried out to observe this effect if exists. Filtering the pairwise similarities obtained using FuzCav fingerprints of the pocket structures resulted in 19 pairs of binding pockets belonging to 13 protein structures that share similarity (Additional file 1: Table S3). Hence, the dataset was split using the binding site similarity to a training dataset ($\sim$83%) containing the thirteen proteins with similar binding pockets and eight more of the proteins with dissimilar binding pockets along with their mutated structures and selected ligands. Finally, the remaining 5 proteins were used as a test dataset ($\sim$17%) along with their corresponding mutated structures and selected ligands. Table 2 shows the proteins included in the train and test sets by binding site similarity.

### Chemical space characterization

**Fig. 2:**
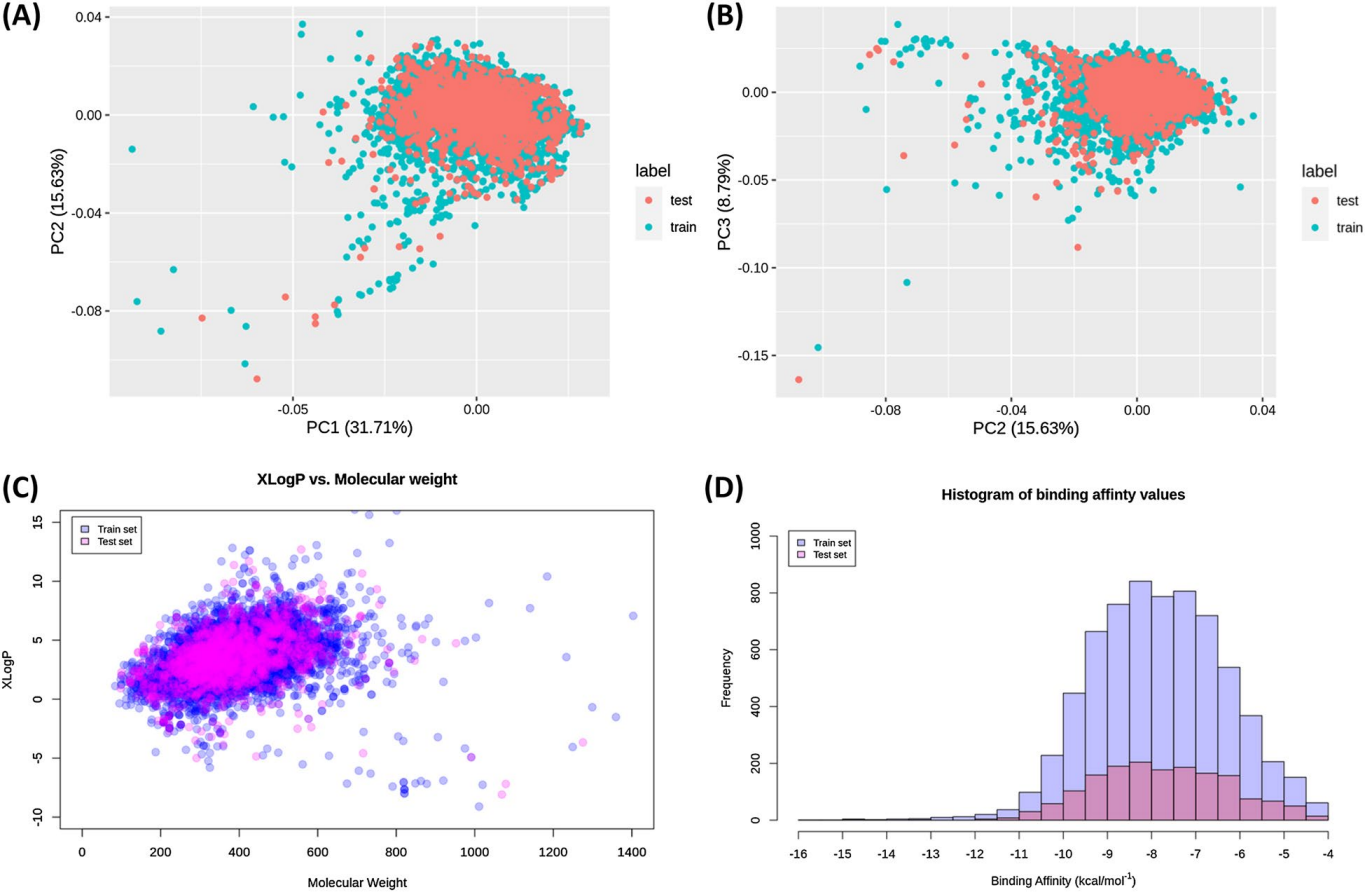
Diversity distribution of ligands in the ligand molecular weight-based data split. Training set (n = 6770) and test set (n = 1651). **A**, **B** Chemical space defined by PCA factorization; **C** chemical space defined by molecular weight as X-axis and XlogP as Y-axis; **D** comparison of binding affinity value distribution in the train/test sets. The blue color stands for the training set, and the red color stands for the test set

**Fig. 3:**
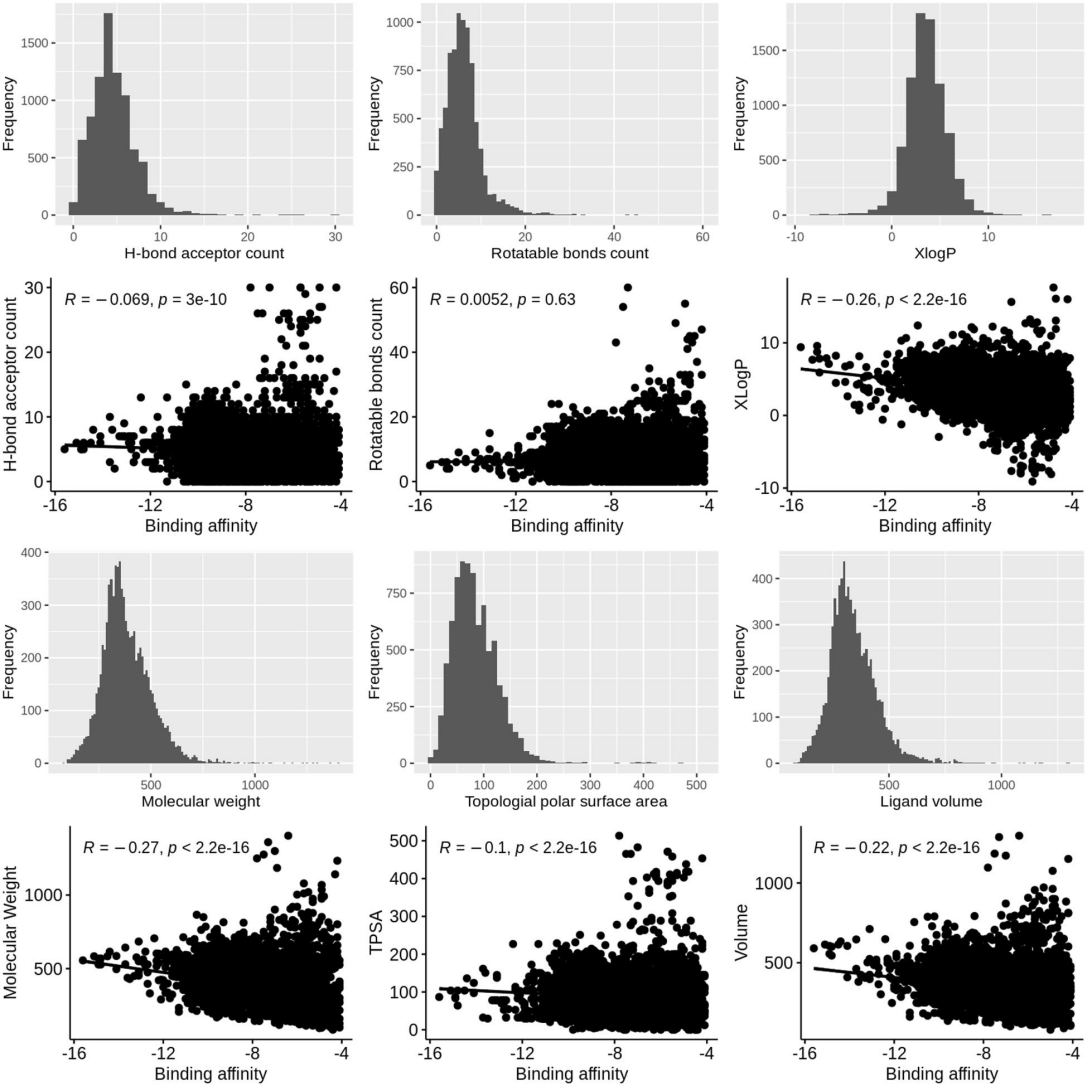
Distributions of six ligand properties and their corresponding correlation to the binding affinity. The six properties from top-left to bottom-right: H-bond acceptor count, rotatable bonds count, XlogP, molecular weight, topological surface area and ligand’s volume

The characterization showed similar results for all data splits. Therefore, one example of the ligand molecular weight-stratified random split is presented in the section. The remaining characterization results are available as Additional file (Additional fie 1: Figs. S1–S5). As shown in Fig. 2, the chemical space of the independent test set was roughly within the scope of the training set, and therefore it is possible to reliably predict the binding affinity in the test set using a machine learning model trained on the training set. Figure 3 shows the distributions of six molecular properties of the ligands in the dataset. These included molecular weight (MW), H-bond acceptor count, rotatable bonds count, octanol-water partitioning coefficient (XlogP), topological polar surface area (TPSA), and Van der Waals volume. It was observed that 90% of the selected ligands contained a maximum of eight hydrogen bond acceptors and ten rotatable bonds without showing a correlation to the binding affinity. XlogP showed a relatively stronger negative correlation to binding affinity (R = $-$ 0.26) where higher XlogP values correspond to smaller binding affinity values (i.e. stronger binding affinity) and 90% of the compounds had a value below 6.029. Similarly, 90% of the compounds in the dataset had a molecular weight smaller than 530 daltons, and the correlation analysis showed a relatively high negative correlation to binding affinity (R = $-$ 0.27). The number of hydrogen bond acceptors and TPSA are usually used to represent hydrophilicity, and as shown in Fig. 3, they had no correlations to the binding affinity (R = $-$  0.069 and $-$ 0.1) than those related to hydrophobicity (XlogP). The ligand volume accounts for the size or bulk of a molecule, and it had a higher correlation than TPSA but lower than molecular weight (R = $-$ 0.22). Apparently, no single descriptor showed a high correlation to binding affinity, and therefore binding affinity could not be reliably predicted from only a single or several molecular descriptors. We hypothesize that combining features representing the protein, the binding site, and the ligand could increase the ability to predict the binding affinity.

### Machine learning modeling

**Fig. 4:**
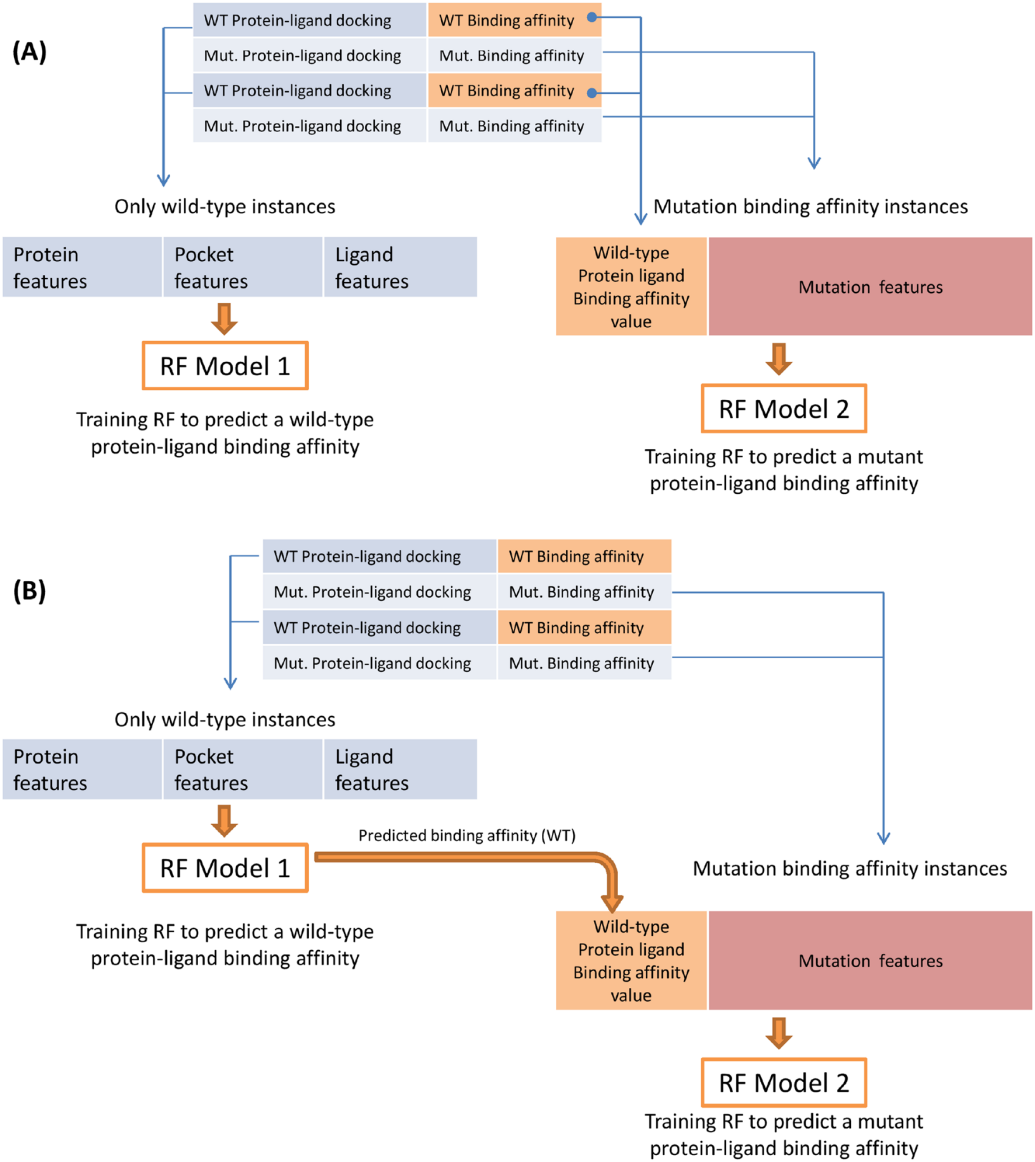
A machine learning model design to predict protein-ligand binding affinity with a single mutation in the protein’s binding site. The design is composed of two models, the first predicts wild-type protein-ligand binding affinity, while the second one predicts the the binding affinity for a mutated protein. Two training scenarios were conducted. **A** The second model was trained using real wild-type protein-ligand binding affinities. **B** The second model was trained using the predicted output of the first model

The architecture in (Fig. 4) shows the proposed model which is composed of two regression models. The first model learns the wild-type protein-ligand binding affinity using protein, binding site, and ligand features only. The second model learns the mutated protein-ligand binding affinity using the wild-type binding affinity and the mutation features. In the second model, the wild-type binding affinity can be obtained either from the real data (docking experiments) or from the output of the first model (predicted wild-type binding affinity) as in Fig. 4A and B respectively. By design, four possible training/testing scenarios were explored for the second model (mutated protein-ligand binding affinity prediction) as follows:

- Training using the real WT binding affinities and testing also using the real data.
- Training using the real data and testing with the predicted data from the first model.
- Training using the predicted data and testing with the real data.
- Training using the predicted output of the first RF model and testing using the predicted data.

#### Protein-ligand binding affinity prediction model

Four machine learning models were trained on six data splits to predict protein-ligand binding affinity using protein, ligand and binding site features. The nested cross validation results (Table 3) shows a good performance of random forest over the other three models: decision tree, lasso regression and ridge regression. These results were expected since random forest [84] has been consistently among the top-performing methods for several bioinformatics tasks [85–87]. Moreover, it has been shown to outperform other feature-based supervised learning approaches in bioinformatics and other domains [88–91]. Random forest models obtained an R^2^ between 0.85 and 0.87 across the six data splits, ridge regression models obtained an R^2^ of 0.76$-$ 0.77, decision tree models obtained an R^2^ of 0,71–0,77 and Lasso regression models obtained an R^2^ of 0.62$-$ 0.65. The tuned parameters and their optimal values for all the models are available in the Additional files (Additional file 1: Tables S4–S7). The best performing random forest models had the following tuned parameter values: number of trees of 500, maximum features of 62 (50% of the total number of features), min_samples_leaf of 1 and min_samples_split of 2.

**Table 3:** Nested cross-validation results of four machine learning models on six data splits to predict protein-ligand binding affinity (Model 1)

Dataset	Random forest	Decision tree	Lasso regression	Ridge regression
BASR	0.85	0.75	0.62	0.76
BSS	0.87	0.77	0.65	0.77
PS	0.86	0.74	0.64	0.76
LS	0.87	0.75	0.62	0.76
LWSR	0.85	0.72	0.62	0.76
LVSR	0.85	0.71	0.62	0.76

The models are: Random Forest, Decision Tree, Lasso Regression and Ridge Regression. The models performance metric reported is correlation coefficient R^2^

Data splits acronyms: *BASR* binding affinity-stratified random split, *PS* protein similarity-based split, *BSS* binding site similarity-based split, *LS* ligand similarity-based split, *LWSR* ligand weight-stratified random split, and *LVSR* ligand volume-stratified random split

The best performing models and their tuned parameter in the nested cross-validation were further validated with the independent test sets of the six data splits as described in the methods section. Table 4 shows the 5-fold cross-validation and test set validation results for the resulting six random forest models. The models trained on stratified data splits (binding affinity, ligand’s molecular weight and ligand’s volume) outperformed the other three models by a substantial margin when evaluated using an independent test set. All models performed exceptionally well in 5-fold cross-validation showing an (R^2^) value higher than 0.84 and RMSE values lower than 0.59. For validation with independent test sets, the model trained on protein similarity-based data split showed the worst performance with a very low R^2^ of 0.25 and an RMSE of 1.06 kcal/mol^-1^. That suggests that the model does not perform well on totally new targets with low to no similarity to the ones it already trained on.

**Fig. 5:**
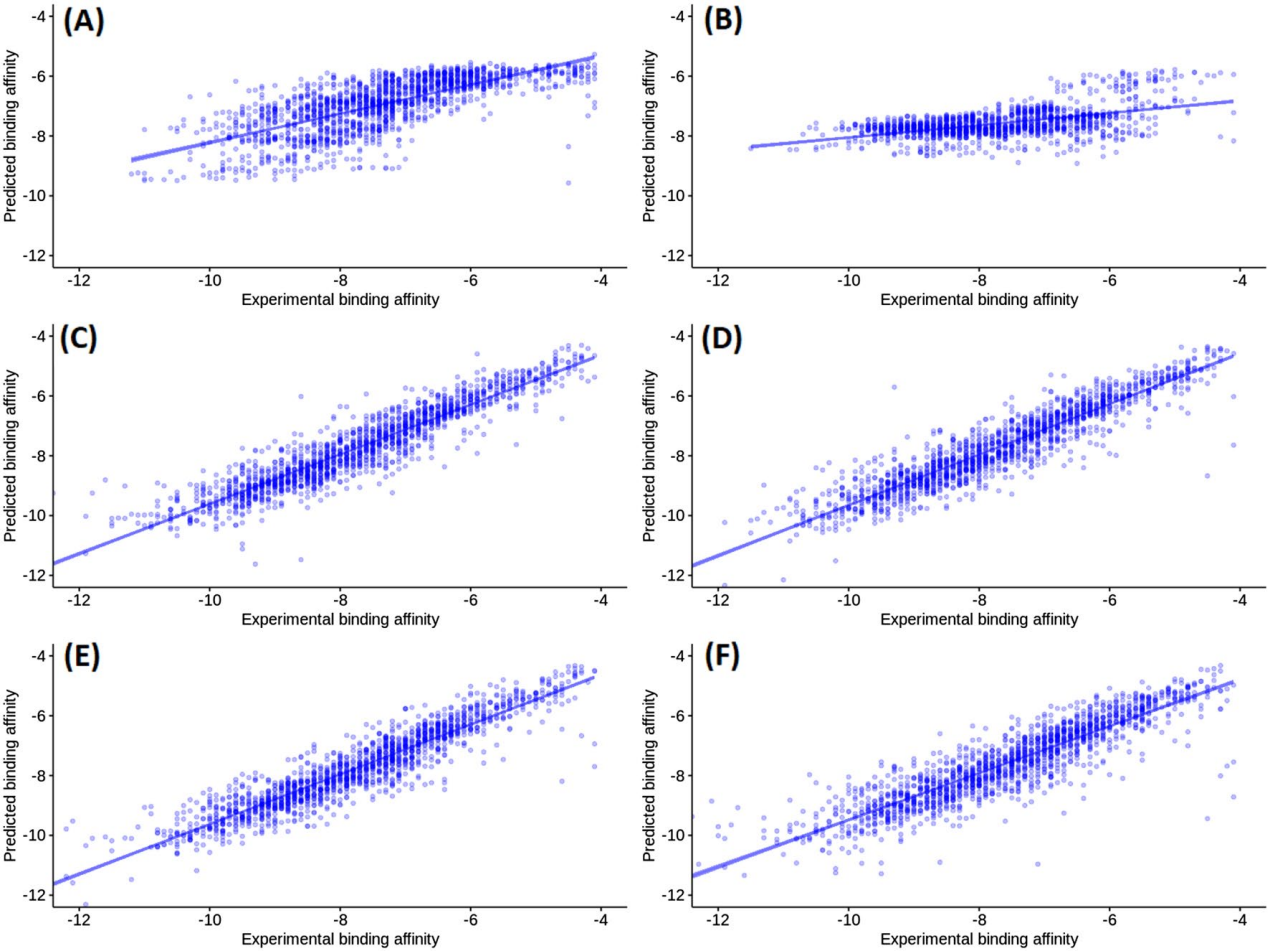
Test set observed versus predicted binding affinities for the trained random forest model on six different split datasets. **A** Binding affinity-stratified random split. **B** Protein similarity-based split. **C** Pocket similarity-based split. **D** Ligand weight-stratified random split. **E** Ligand volume-stratified random split. **F** Ligand similarity-based split

Furthermore, the model trained on binding site similarity-based data split also had a bad performance with R^2^ of 0.42 and RMSE of 1.0 kcal/mol^-1^. However, this is slightly better than the protein similarity-based model. That indicates that the binding site similarity is important to predict the binding affinity. It also suggests that this model is not applicable for new protein targets that are structurally dissimilar to have a dissimilar binding site to the ones in PSnpBind. The model trained on ligand similarity-based data split showed a good performance with R^2^ of 0.81 and RMSE of 0.68 kcal/mol^-1^. The diversity of the ligands between the train and test datasets did not largely affect the ability to predict the protein-ligand binding affinity which suggests that such a model can be used to predict new instances with dissimilar ligands and still achieve a good performance. Furthermore, the models trained on stratified data splits showed almost identical evaluation metrics with an R^2^ of 0.87 and RMSE of 0.55$-$−0.56 kcal/mol^-1^. Considering that, the model trained on the ligand weight stratified data split was selected for downstream analysis of Y-randomization, prediction time evaluation and feature importance. Figure 5 shows a scatter plot of the measured against the predicted mutated protein-ligand binding affinity values resulting from training a random forest model using six different data splits. The prediction speed evaluation showed that the obtained models are capable of predicting 10k instances per second. Hence, when compared to the time required on average for a single docking in PSnpBind ( 60 s), this approach is faster by five orders of magnitude.

A third approach was applied to validate the best performance model, y-randomization, a tool used to test whether the predictions obtained by the model are made by chance or not. The Y-randomization validation method was applied ten times with test set validation and returned an R^2^ of zero value each time. The results were conclusive that the y-randomized models were significantly worse, and the obtained models had a high performance that is not related to chance.

**Table 4:** Training/testing results of a random forest model that uses features of proteins, ligands and binding sites to predict the wild-type protein-ligand binding affinity

Metric	BASR	PS	BSS	LS	LWSR	LVSR
R^2^ (5-fold CV)	0.85	0.87	0.86	0.87	0.85	0.85
MAE (5-fold CV)	0.42	0.41	0.41	0.40	0.42	0.42
MSE (5-fold CV)	0.34	0.32	0.33	0.30	0.33	0.34
RMSE (5-fold CV)	0.58	0.56	0.57	0.55	0.58	0.58
R^2^ (test set)	0.87	0.25	0.42	0.81	0.87	0.87
MAE (test set)	0.40	0.86	0.78	0.48	0.41	0.40
MSE (test set)	0.30	1.12	1.01	0.46	0.31	0.31
RMSE (test set)	0.55	1.06	1.00	0.68	0.56	0.56
RAE (test set)	0.33	0.85	0.75	0.39	0.33	0.33
RRSE (test set)	0.37	0.87	0.77	0.44	0.37	0.36

The model was trained/tested against six different data splits: *BASR* binding affinity-stratified random split, *PS* protein similarity-based split, *BSS* binding site similarity-based split, *LS* ligand similarity-based split, *LWSR* ligand weight-stratified random split, and *LVSR* ligand volume-stratified random split

#### Mutated protein-ligand binding affinity prediction model

The second model uses features of mutations besides either a real or predicted wild-type protein-ligand binding affinity to predict the mutated protein-ligand binding affinity. Table 5 shows the 5-fold cross-validation and test set validation results for the resulting four random forest models. It appears from the table that when training the model with real wild-type protein-ligand binding affinities (i.e. obtained with docking) and testing it with real data also, it achieves the best performance with a determination coefficient (R^2^ = 0.90 and RMSE = 0.50 kcal/mol^-1^). The same model (trained with real data) resulted in slightly less but very close performance when tested with predicted wild-type protein-ligand binding affinity values (R^2^ = 0.89 and RMSE = 0.52 kcal/mol^-1^).

**Fig. 6:**
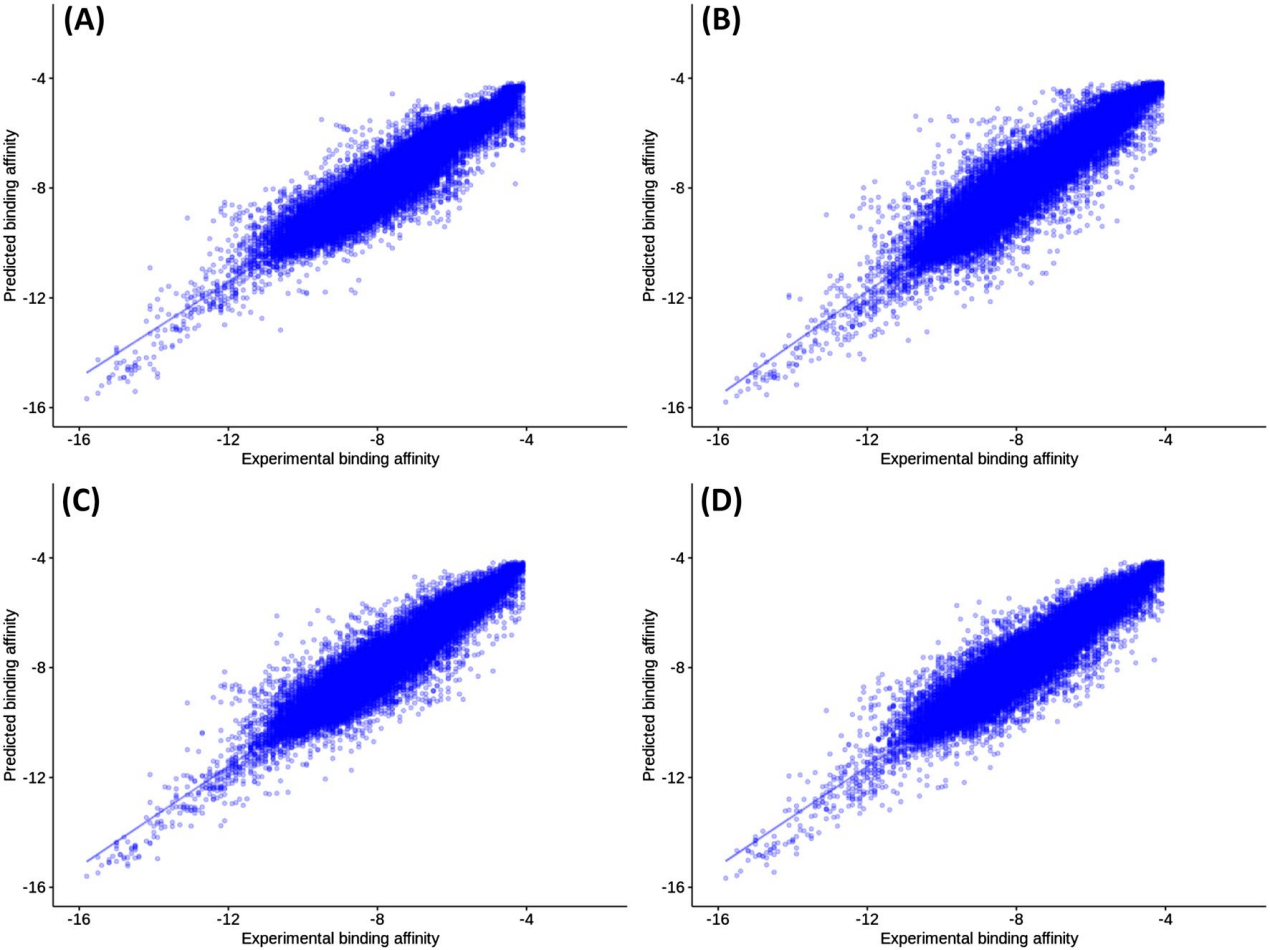
Test set observed versus predicted binding affinities to mutated proteins using two trained random forest models (one using measured wild-type protein-ligand binding affinity and the second using predicted wild-type protein-ligand binding affinity as input). **A** The model trained with measured wild-type binding affinity and tested using measured wild-type binding affinity. **B** The model trained with measured wild-type binding affinity and tested using predicted wild-type binding affinity. **C** The model trained with predicted wild-type binding affinity and tested using measured wild-type binding affinity. **D** The model trained with predicted wild-type binding affinity and tested using predicted wild-type binding affinity

Meanwhile, the model trained with predicted wild-type protein-ligand binding affinity (pred-real in Table 5) showed the lowest performance compared to the other models when tested with real wild-type protein-ligand binding affinities (R^2^ = 0.87 and RMSE = 0.56 kcal/mol^-1^). However, the results get slightly better when tested with predicted wild-type protein-ligand binding affinity data (R^2^ = 0.88 and RMSE = 0.52 kcal/mol^-1^). Figure 6 shows a scatter plot of the measured against the predicted mutated protein-ligand binding affinity values resulting from training a random forest model using four train/test scenarios. Even though, the four models performed relatively well and achieved an R^2^ above 0.86 and an RMSE below 0.56 kcal/mol^-1^. Regarding prediction speed, the obtained models need 0.1 ms to predict a single instance which is faster by five orders of magnitude than the average time needed for a single docking in PSnpBind.

**Table 5:** Training/testing results of a random forest model that uses features of mutations besides either a real or predicted wild-type protein-ligand binding affinity to predict the mutated protein-ligand binding affinity

Metric	real–real	real-pred	pred-real	pred–pred
R^2^ (5-fold CV)	0.90	0.90	0.89	0.89
MAE (5-fold CV)	0.35	0.35	0.38	0.38
MSE (5-fold CV)	0.25	0.25	0.28	0.28
RMSE (5-fold CV)	0.50	0.50	0.53	0.53
R^2^ (test set)	0.90	0.89	0.87	0.88
MAE (test set)	0.35	0.38	0.40	0.38
MSE (test set)	0.25	0.27	0.32	0.28
RMSE (test set)	0.50	0.52	0.56	0.52
RAE (test set)	0.28	0.30	0.32	0.30
RRSE (test set)	0.33	0.34	0.37	0.34

The model was trained/tested in four scenrios. real–real: the model trained using real wild-type protein-ligand bindinf affinity data and tested on real binding affinities also. real-pred: the model is trained using real wild-type protein-ligand binding affinity data and tested on predicted binding affinities. pred-real: the model is trained using predicted wild-type protein-ligand binding affinity data and tested on real binding affinities. pred–pred: the model is trained using predicted wild-type protein-ligand binding affinity data and tested on predicited binding affinities

### Comparison with publicly available models

The approach proposed in this work is composed of two models. Since the first ML model (predicting wild-type protein-ligand binding affinity) does not contain mutation information, it can be compared to similar models from the literature designed to predict protein-ligand binding affinity as described in the related work section. This model outperformed the reported performances by [31] which obtained an R^2^ of 0.73. Also, it performed better than the DeepAtom Convolutional Neural Networks (CNN) deep learning model (2019) [92] which reported a Pearson’s correlation of 0.83 (R^2^ = 0.69). The authors claimed that the model outperforms the recent state-of-the-art models in predicting protein-ligand binding affinity like Kdeep [93] and DeepSite [94]. Even though it is hard to compare regression models without applying them to the same dataset and using the same evaluation techniques, the DeepAtom model was trained and tested on the PDBbind core set 2016 which is the same dataset that PSnpBind, the primary data source of this work, was built upon. However, the authors used the entire core set since they were not interested in human variants only as in our case.

The second ML model (predicting mutated protein-ligand binding affinity) showed the best performance when trained using measured wild-type protein-ligand binding affinity data besides the mutation features. This model reported an (R^2^ = 0.89) which is higher than what was reported by the mCSM-lig method in the best case scenario (Pearson correlation R = 0.737, R^2^ = 0.543) [34] and the mCSM-AB method (Pearson correlation R = 0.53) [35]. Besides comparisons with other machine learning approaches, the obtained models in this study outperformed a method developed in 2018 that uses free energy calculations (Rosetta + molecular dynamics MD) to estimate ligand-binding affinity changes upon mutation and they applied it to 134 mutations [95]. The method reported an RMSE of 1.2 kcal/mol^-1^ for the full benchmark set while the model developed in this work achieved an RMSE of 0.50-.56 kcal/mol^-1^.

### Feature importance

**Fig. 7:**
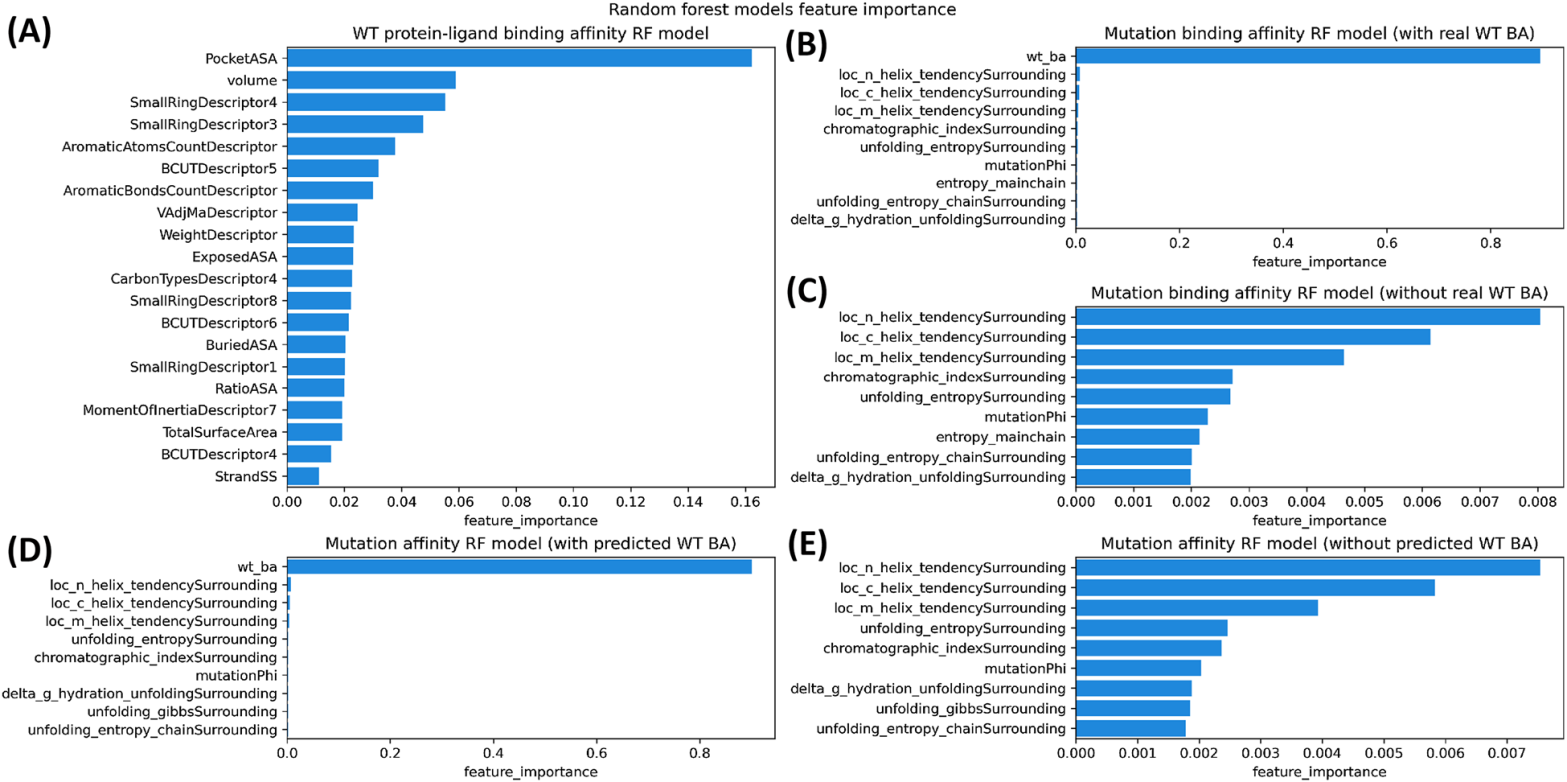
Feature importance of random forest models: **A** important features for WT BA prediction model; **B** important features for mutation BA prediction model trained with real WT BA; **C** same as B but with wt_ba feature removed to better visualize the other features; **D** important features for mutation BA prediction model trained with predicted WT BA; **E** same as D but with wt_ba feature removed to better visualize the other features

Feature importance analysis was performed on the two RF models obtained for WT and mutated protein-ligand binding affinity respectively. Figure 7 shows the most important features of each model. Figure 7A shows the most important features of the first model predicting the WT protein-ligand binding affinity. The volume and the accessible surface area (ASA) of the binding site are the top most influential features on binding affinity prediction. Other related features to the binding site ASA appeared in the list like the number of buried and exposed residues. Among the most ligand-related features contributing to predicting the binding affinity is small ring descriptors followed by other types of descriptors like aromatics bonds and atom counts, molecular weight, carbon types, total surface area and moment of inertia. Clearly, 1D, 2D and 3D characteristics of the ligand are important to predict the binding affinity. The highlighted features of the ligand and binding site align well with the known relation between ligands and binding sites. For example, a large volume ligand will probably not bind to a small binding site since it will not fit inside it. Figure 7B and C show the important features to the model predicting the mutated protein-ligand binding affinity which is trained on real WT binding affinity data. As one would expect, the WT protein-ligand binding affinity was the most important feature for prediction by a large margin. Therefore, in Fig. 7C, that feature was removed to show more clearly the contribution of other features. The properties of the surrounding of the mutated amino acid were at the top of the list. The local environment of the mutation captures the physicochemical information of an area that is close to the ligand where a change could affect the binding affinity. Moreover, the phi dihedral angle of the mutated amino acid also appeared in the list. Figure 7D and E show the important features to the model predicting the mutated protein-ligand binding affinity which is trained on predicted WT binding affinity data. Similar to the previous model (trained on real WT BA data), the WT binding affinity was the most important feature and nine out of ten features in the list were the same.

## Conclusion

Machine learning models can be applied to predict the protein-ligand binding affinity for proteins with single-point mutations in their binding sites with high accuracy. This study provides an evaluation of six data split scenarios to obtain the best models and concluded that data split by ligand molecular weight, ligand volume and binding affinity resulted in the best-performance ML models. Moreover, it showed that the models perform best when tested on structurally similar proteins or at least proteins with structurally similar binding pockets while their performance significantly degrades when tested on structurally different proteins or binding pockets. Furthermore, the study showed the importance of features that capture the binding site volume and accessible surface area and ligands’ 2D and 3D descriptors on the prediction of binding affinity. Additionally, features that capture the physicochemical properties of the residues surrounding the mutation’s amino acid are important to predict the ligand’s binding affinity to the protein with a single-point mutation in its binding site. We report an improvement in the prediction performance of the ML models, compared to several published models developed for protein-ligand binding affinity prediction. The obtained models have high accuracy and a much higher speed than conventional docking approaches (five orders of magnitude) which makes it feasible to be used as a complementary method in early-stage drug discovery. It can be applied to obtain a better overview of the ligand’s binding affinity changes across protein variants carried by people in the population and to select potential leads that achieve a better affinity overall protein variants.

## Supplementary Information

**Additional file 1:** **Table S1.** Protein pairwise sequence similarity for PSnpBind proteins (26 in total). **Table S2.** HMMER search results against Pfam for PSnpBind proteins (26 in total). **Table S3.** Pairwise binding pocket similarity scores for the 26 proteins in descending ordered by score. The similarity was calculated from the fingerprints generated using FuzCav. The table contains only similar pockets with a similarity score > 0.16 (as mentioned in Weill et al. [70]). **Table S4.** Optimal parameters for Random Forest models trained on six data splits using nested cross-validation. Data splits acronyms: BASR: Binding affinity-stratified random split, PS: Protein similarity-based split, BSS: Binding site similarity-based split, LS: Ligand similarity-based split, LWSR: Ligand weight-stratified random split, and LVSR: Ligand volume-stratified random split. **Table S5.** Optimal parameters for Decision Tree models trained on six data splits using nested cross-validation. Data splits acronyms: BASR: Binding affinity-stratified random split, PS: Protein similarity-based split, BSS: Binding site similarity-based split, LS: Ligand similarity-based split, LWSR: Ligand weight-stratified random split, and LVSR: Ligand volume-stratified random split. **Table S6.** Optimal parameters for Lasso Regression models trained on six data splits using nested cross-validation. Data splits acronyms: BASR: Binding affinity-stratified random split, PS: Protein similarity-based split, BSS: Binding site similarity-based split, LS: Ligand similarity-based split, LWSR: Ligand weight-stratified random split, and LVSR: Ligand volume-stratified split. **Table S7.** Optimal parameters for Ridge Regression models trained on six data splits using nested cross-validation. Data splits acronyms: BASR: Binding affinity-stratified random split, PS: Protein similarity-based split, BSS: Binding site similarity-based split, LS: Ligand similarity-based split, LWSR: Ligand weight-stratified random split, and LVSR: Ligand volume-stratified split.** Figure S1.** Diversity distribution of ligands in the random data split stratified on binding affinity. (A, B) Chemical space defined by PCA factorization; (C) chemical space defined by molecular weight as X-axis and XlogP as Y. **Figure S2.** Diversity distribution of ligands in the protein similarity-based data split. (A, B) Chemical space defined by PCA factorization; (C) chemical space defined by molecular weight as X-axis and XlogP as Y. **Figure S3.** Diversity distribution of ligands in the binding pocket similarity-based data split. (A, B) Chemical space defined by PCA factorization; (C) chemical space defined by molecular weight as X-axis and XlogP as Y. **Figure S4.** Diversity distribution of ligands in the ligand similarity-based data split. (A, B) Chemical space defined by PCA factorization; (C) chemical space defined by molecular weight as X-axis and XlogP as Y. **Figure S5.** Diversity distribution of ligands in the in the random data split stratified on ligand volume. (A, B) Chemical space defined by PCA factorization; (C) chemical space defined by molecular weight as X-axis and XlogP as Y.

## Data Availability

All PSnpBind-ML data and code to train and evaluate the models are freely available without any restriction. The PSnpBind dockings dataset can be downloaded from Zenodo https://doi.org/10.5281/zenodo.6968470. The code used to produce the machine learning models reported in this work is available on GitHub https://github.com/BiGCAT-UM/PSnpBind-ML-notebook and the notebooks are also available through MyBinder for reproducibility https://mybinder.org/v2/gh/ammar257ammar/PSnpBind-ML-notebook/ccde6b4f84f9d1b9425b3fc271ddf72f91dca17c. The pretrained model obtained in this work are available via Zenodo https://doi.org/10.5281/zenodo.6972486

## Abbreviations

ML: Machine learning
RF: Random forest
WT: Wild type
SNP: Single-nucleotide polymorphism
DSRI: Data Science Research Infrastructure
CDK: Chemistry Development Kit
SOCN: Sequence-order-coupling number
