# Supplementary material for: PSnpBind-ML: predicting the effect of binding site mutations on protein-ligand binding affinity
Source: J Cheminform. 2023 Mar 2;15:31. doi: 10.1186/s13321-023-00701-3 (PMC9983232; doi:10.1186/s13321-023-00701-3)
Supplement: Supplementary file 1 — Additional file 1: Table S1. Protein pairwise sequence similarity for PSnpBind proteins (26 in total). Table S2. HMMER search results against Pfam for PSnpBind proteins (26 in total). Table S3. Pairwise binding pocket similarity scores for the 26 proteins in descending ordered by score. The similarity was calculated from the fingerprints generated using FuzCav. The table contains only similar pockets with a similarity score > 0.16 (as mentioned in Weill et al. [70]). Table S4. Optimal parameters for Random Forest models trained on six data splits using nested cross-validation. Data splits acronyms: BASR: Binding affinity-stratified random split, PS: Protein similarity-based split, BSS: Binding site similarity-based split, LS: Ligand similarity-based split, LWSR: Ligand weight-stratified random split, and LVSR: Ligand volume-stratified random split. Table S5. Optimal parameters for Decision Tree models trained on six data splits using nested cross-validation. Data splits acronyms: BASR: Binding affinity-stratified random split, PS: Protein similarity-based split, BSS: Binding site similarity-based split, LS: Ligand similarity-based split, LWSR: Ligand weight-stratified random split, and LVSR: Ligand volume-stratified random split. Table S6. Optimal parameters for Lasso Regression models trained on six data splits using nested cross-validation. Data splits acronyms: BASR: Binding affinity-stratified random split, PS: Protein similarity-based split, BSS: Binding site similarity-based split, LS: Ligand similarity-based split, LWSR: Ligand weight-stratified random split, and LVSR: Ligand volume-stratified split. Table S7. Optimal parameters for Ridge Regression models trained on six data splits using nested cross-validation. Data splits acronyms: BASR: Binding affinity-stratified random split, PS: Protein similarity-based split, BSS: Binding site similarity-based split, LS: Ligand similarity-based split, LWSR: Ligand weight-stratified random split, and LVSR: Ligand volum [file 13321_2023_701_MOESM1_ESM.pdf]

PSnpBind-ML: predicting the effect of binding site mutations on protein-ligand binding affinity

Ammar Ammar[1], Chris Evelo[1], Rachel Cavill[2], Egon Willighagen[1]

[1]Department of Bioinformatics—BiGCaT, NUTRIM, Maastricht University, The Netherlands

[2]Department of Data Science and Knowledge Engineering, Maastricht University, The Netherlands

Additional file Tables

Table S1. Protein pairwise sequence similarity for PSnpBind proteins (26 in total).

Protein A	Protein B	E-value	Identity
sp|P00734|THRB_HUMAN	sp|P00742|FA10_HUMAN	7.64E-20	50.562
sp|P00742|FA10_HUMAN	sp|P00734|THRB_HUMAN	6.99E-21	47.573
sp|P37231|PPARG_HUMAN	sp|P10275|ANDR_HUMAN	1.37E-13	44.286
sp|P00734|THRB_HUMAN	sp|P00742|FA10_HUMAN	5.85E-52	39.615
sp|P00742|FA10_HUMAN	sp|P00734|THRB_HUMAN	4.73E-52	39.615
sp|P00519|ABL1_HUMAN	sp|Q08881|ITK_HUMAN	5.53E-97	37.528
sp|Q08881|ITK_HUMAN	sp|P00519|ABL1_HUMAN	3.68E-97	37.528
sp|P24941|CDK2_HUMAN	sp|Q16539|MK14_HUMAN	1.91E-61	37.458
sp|Q16539|MK14_HUMAN	sp|P24941|CDK2_HUMAN	2.31E-61	37.458
sp|P00734|THRB_HUMAN	sp|P03951|FA11_HUMAN	9.22E-36	36.434
sp|P03951|FA11_HUMAN	sp|P00734|THRB_HUMAN	9.27E-36	36.434
sp|P00519|ABL1_HUMAN	sp|P23458|JAK1_HUMAN	5.07E-44	35.273
sp|P23458|JAK1_HUMAN	sp|P00519|ABL1_HUMAN	2.62E-44	35.273
sp|O60674|JAK2_HUMAN	sp|P00519|ABL1_HUMAN	5.18E-48	35.192
sp|P00519|ABL1_HUMAN	sp|O60674|JAK2_HUMAN	7.97E-48	35.192
sp|P00742|FA10_HUMAN	sp|P03951|FA11_HUMAN	2.36E-38	34.043
sp|P03951|FA11_HUMAN	sp|P00742|FA10_HUMAN	3.73E-38	34.043
sp|P03372|ESR1_HUMAN	sp|P37231|PPARG_HUMAN	3.72E-26	33.032
sp|P37231|PPARG_HUMAN	sp|P03372|ESR1_HUMAN	2.89E-26	33.032
sp|O14757|CHK1_HUMAN	sp|P24941|CDK2_HUMAN	5.73E-30	33
sp|P24941|CDK2_HUMAN	sp|O14757|CHK1_HUMAN	3.59E-30	33
sp|P00749|UROK_HUMAN	sp|P03951|FA11_HUMAN	9.17E-37	32.54

sp|P03951|FA11_HUMAN	sp|P00749|UROK_HUMAN	2.19E-36	32.54
sp|O60674|JAK2_HUMAN	sp|P24941|CDK2_HUMAN	1.97E-23	32.512
sp|P24941|CDK2_HUMAN	sp|O60674|JAK2_HUMAN	5.96E-24	32.512
sp|O14757|CHK1_HUMAN	sp|P23458|JAK1_HUMAN	7.81E-17	31.343
sp|P23458|JAK1_HUMAN	sp|O14757|CHK1_HUMAN	1.68E-16	31.343
sp|P23458|JAK1_HUMAN	sp|P24941|CDK2_HUMAN	5.07E-24	31.068
sp|P24941|CDK2_HUMAN	sp|P23458|JAK1_HUMAN	1.40E-24	31.068
sp|O14757|CHK1_HUMAN	sp|O60674|JAK2_HUMAN	6.20E-15	30.732
sp|O60674|JAK2_HUMAN	sp|O14757|CHK1_HUMAN	1.51E-14	30.732
sp|O60674|JAK2_HUMAN	sp|Q16539|MK14_HUMAN	7.10E-18	30.583
sp|Q16539|MK14_HUMAN	sp|O60674|JAK2_HUMAN	2.30E-18	30.583
sp|O14757|CHK1_HUMAN	sp|Q16539|MK14_HUMAN	7.46E-20	30.256
sp|Q16539|MK14_HUMAN	sp|O14757|CHK1_HUMAN	5.64E-20	30.256
sp|P23458|JAK1_HUMAN	sp|Q08881|ITK_HUMAN	8.58E-35	30.142
sp|Q08881|ITK_HUMAN	sp|P23458|JAK1_HUMAN	5.82E-35	30.142
sp|P24941|CDK2_HUMAN	sp|Q08881|ITK_HUMAN	3.51E-18	29.557
sp|Q08881|ITK_HUMAN	sp|P24941|CDK2_HUMAN	7.30E-18	29.557
sp|O60674|JAK2_HUMAN	sp|Q08881|ITK_HUMAN	9.02E-36	29.492
sp|Q08881|ITK_HUMAN	sp|O60674|JAK2_HUMAN	5.75E-36	29.492
sp|O14965|AURKA_HUMAN	sp|P24941|CDK2_HUMAN	3.58E-37	29.47
sp|P24941|CDK2_HUMAN	sp|O14965|AURKA_HUMAN	2.64E-37	29.47
sp|O14965|AURKA_HUMAN	sp|Q16539|MK14_HUMAN	3.72E-25	29.07
sp|Q16539|MK14_HUMAN	sp|O14965|AURKA_HUMAN	3.32E-25	29.07
sp|O14965|AURKA_HUMAN	sp|P11309|PIM1_HUMAN	1.02E-30	28.966
sp|P11309|PIM1_HUMAN	sp|O14965|AURKA_HUMAN	7.94E-31	28.966
sp|P00519|ABL1_HUMAN	sp|P24941|CDK2_HUMAN	1.26E-20	28.634
sp|P24941|CDK2_HUMAN	sp|P00519|ABL1_HUMAN	3.23E-21	28.634
sp|O14757|CHK1_HUMAN	sp|O14965|AURKA_HUMAN	1.22E-33	28.571
sp|O14965|AURKA_HUMAN	sp|O14757|CHK1_HUMAN	1.03E-33	28.571
sp|P23458|JAK1_HUMAN	sp|Q16539|MK14_HUMAN	1.59E-15	28.571
sp|Q16539|MK14_HUMAN	sp|P23458|JAK1_HUMAN	5.05E-16	28.571
sp|P11309|PIM1_HUMAN	sp|P24941|CDK2_HUMAN	8.95E-20	28.512
sp|P24941|CDK2_HUMAN	sp|P11309|PIM1_HUMAN	8.52E-20	28.512
sp|P00519|ABL1_HUMAN	sp|Q16539|MK14_HUMAN	3.99E-20	28.511
sp|Q16539|MK14_HUMAN	sp|P00519|ABL1_HUMAN	7.83E-21	28.511
sp|P10275|ANDR_HUMAN	sp|P03372|ESR1_HUMAN	1.43E-45	28.291

sp|P00734|THRB_HUMAN	sp|P00749|UROK_HUMAN	2.23E-30	28.029
sp|P00749|UROK_HUMAN	sp|P00734|THRB_HUMAN	1.61E-30	28.029
sp|P03372|ESR1_HUMAN	sp|P10275|ANDR_HUMAN	4.82E-43	27.731
sp|O14965|AURKA_HUMAN	sp|P23458|JAK1_HUMAN	4.86E-17	27.638
sp|P23458|JAK1_HUMAN	sp|O14965|AURKA_HUMAN	1.33E-16	27.638
sp|O14757|CHK1_HUMAN	sp|P00519|ABL1_HUMAN	5.15E-13	27.536
sp|P00519|ABL1_HUMAN	sp|O14757|CHK1_HUMAN	1.32E-12	27.536
sp|P11309|PIM1_HUMAN	sp|Q08881|ITK_HUMAN	8.50E-17	27.273
sp|Q08881|ITK_HUMAN	sp|P11309|PIM1_HUMAN	1.68E-16	27.273
sp|O60674|JAK2_HUMAN	sp|Q08881|ITK_HUMAN	4.05E-23	27.174
sp|Q08881|ITK_HUMAN	sp|O60674|JAK2_HUMAN	2.69E-23	27.174
sp|P00742|FA10_HUMAN	sp|P00749|UROK_HUMAN	3.67E-31	26.942
sp|P00749|UROK_HUMAN	sp|P00742|FA10_HUMAN	1.05E-31	26.773
sp|O60674|JAK2_HUMAN	sp|P23458|JAK1_HUMAN	2.85E-22	26.733
sp|P23458|JAK1_HUMAN	sp|O60674|JAK2_HUMAN	2.52E-22	26.733
sp|O60674|JAK2_HUMAN	sp|P00519|ABL1_HUMAN	2.75E-24	26.545
sp|P00519|ABL1_HUMAN	sp|O60674|JAK2_HUMAN	5.57E-24	26.545
sp|P00519|ABL1_HUMAN	sp|P11309|PIM1_HUMAN	3.59E-16	26.459
sp|P11309|PIM1_HUMAN	sp|P00519|ABL1_HUMAN	1.11E-16	26.459
sp|O14965|AURKA_HUMAN	sp|O60674|JAK2_HUMAN	2.59E-21	26.296
sp|P00519|ABL1_HUMAN	sp|P23458|JAK1_HUMAN	1.36E-20	26.296
sp|P23458|JAK1_HUMAN	sp|P00519|ABL1_HUMAN	8.06E-21	26.296
sp|O14965|AURKA_HUMAN	sp|P00519|ABL1_HUMAN	1.32E-24	26.154
sp|P00519|ABL1_HUMAN	sp|O14965|AURKA_HUMAN	3.31E-24	26.154
sp|P23458|JAK1_HUMAN	sp|Q08881|ITK_HUMAN	3.19E-21	26.009
sp|Q08881|ITK_HUMAN	sp|P23458|JAK1_HUMAN	2.02E-21	26.009
sp|O60674|JAK2_HUMAN	sp|O14965|AURKA_HUMAN	7.01E-21	25.926
sp|O14757|CHK1_HUMAN	sp|P11309|PIM1_HUMAN	3.13E-21	25.818
sp|P11309|PIM1_HUMAN	sp|O14757|CHK1_HUMAN	2.06E-21	25.818
sp|O14965|AURKA_HUMAN	sp|Q08881|ITK_HUMAN	1.31E-22	25.769
sp|Q08881|ITK_HUMAN	sp|O14965|AURKA_HUMAN	2.01E-22	25.769
sp|P10275|ANDR_HUMAN	sp|P37231|PPARG_HUMAN	2.57E-15	25.352
sp|P11309|PIM1_HUMAN	sp|Q16539|MK14_HUMAN	7.25E-17	24.783
sp|Q16539|MK14_HUMAN	sp|P11309|PIM1_HUMAN	8.34E-17	24.783
sp|O14757|CHK1_HUMAN	sp|Q08881|ITK_HUMAN	1.15E-13	24.757
sp|Q08881|ITK_HUMAN	sp|O14757|CHK1_HUMAN	1.50E-13	24.757

sp|P23458|JAK1_HUMAN	sp|O14965|AURKA_HUMAN	7.97E-09	24.453
sp|O14965|AURKA_HUMAN	sp|P23458|JAK1_HUMAN	2.53E-09	23.381
sp|Q08881|ITK_HUMAN	sp|Q16539|MK14_HUMAN	1.43E-12	22.549
sp|Q16539|MK14_HUMAN	sp|Q08881|ITK_HUMAN	8.31E-13	22.549
sp|P23458|JAK1_HUMAN	sp|P24941|CDK2_HUMAN	4.49E-08	21.824
sp|P24941|CDK2_HUMAN	sp|P23458|JAK1_HUMAN	1.15E-08	21.824

Table S2. HMMER search results against Pfam for PSnpBind proteins (26 in total)

Protein Family	UniProt ID	E-value
Androgen_recep	P10275	1E-258
ANF_receptor	P39086	1.4E-80
Ank	Q9H2K2	2E-109
Ank_2	Q9H2K2	7E-109
Ank_3	Q9H2K2	2E-100
Ank_4	Q9H2K2	2E-106
Ank_5	Q9H2K2	2E-113
Asp	P56817	7.3E-48
Astacin	P39900	2.4E-07
BET	O60885	3.1E-32
BRD4_CDT	O60885	6.1E-28
Bromodomain	O60885	1E-45
BTK	Q08881	2.3E-16
Carb_anhydrase	P00918	9E-102
cEGF	P00742	5.4E-07
DSPc	P18031	4.5E-08
DUF1986	P03951	2.6E-07
DUF1986	P00742	2.9E-06
DUF1986	P00734	4.4E-06
DUF316	P00749	3.8E-08
EGF	P00742	1.8E-10
ESR1_C	P03372	4E-26
F_actin_bind	P00519	1.1E-32
FERM_F1	O60674	8.3E-44
FERM_F1	P23458	3.5E-35
FERM_F2	P23458	2.6E-60
FERM_F2	O60674	8.6E-48
FERM_M	O60674	2E-06
FXa_inhibition	P00742	8.1E-12
GAF	Q9Y233	2.9E-31
GAF_2	Q9Y233	9.3E-20

GAF_3	Q9Y233	6.3E-12
Gla	P00742	3.2E-24
Gla	P00734	4.8E-21
Haspin_kinase	P24941	3.1E-08
Haspin_kinase	P00519	1.4E-06
HATPase_c	P07900	3.5E-17
HATPase_c_3	P07900	3.7E-15
hEGF	P00742	2.5E-07
Hemopexin	P39900	6.7E-52
Hormone_recep	P03372	5.7E-37
Hormone_recep	P10275	3E-34
Hormone_recep	P37231	1.6E-19
HSP90	P07900	1E-237
Jak1_Phl	P23458	1.2E-56
Jak1_Phl	O60674	6.4E-25
Kdo	P11309	8E-10
Kdo	Q16539	9.4E-08
Kinase-like	P11309	3E-12
Kinase-like	O14757	9.4E-10
Kinase-like	O14965	2.8E-09
Kinase-like	P24941	1.5E-06
Kinase-like	P23458	2E-06
Kinase-like	O60674	3.2E-06
Kringle	P00734	5E-52
Kringle	P00749	1.7E-25
Lig_chan	P39086	1E-56
Lig_chan-Glu_bd	P39086	2.9E-49
Metallopep	P39900	2.9E-06
Oest_recep	P03372	5.3E-68
P53	P04637	8.2E-64
P53_TAD	P04637	6.9E-15
P53_tetramer	P04637	3.6E-21
PAN_1	P03951	9.3E-37
PAN_4	P03951	1.2E-20
PARP	Q9H2K2	1.6E-28
PDEase_I	Q9Y233	3.3E-75
Peptidase_M10	P39900	3E-64
Peripla_BP_6	P39086	2.2E-10
PG_binding_1	P39900	9.7E-18
PH	Q08881	6.5E-17
PH_8	Q08881	1.1E-06
Pkinase	P24941	1E-81

Pkinase	O60674	3.2E-77
Pkinase	P23458	5.9E-75
Pkinase	O14965	5.2E-73
Pkinase	O14757	2.2E-69
Pkinase	P11309	2E-68
Pkinase	Q16539	5.8E-68
Pkinase	P00519	4.3E-51
Pkinase	Q08881	9.6E-50
Pkinase_fungal	O14757	1.3E-06
Pkinase_Tyr	O60674	7E-151
Pkinase_Tyr	P23458	1E-150
Pkinase_Tyr	P00519	6E-102
Pkinase_Tyr	Q08881	7E-95
Pkinase_Tyr	O14965	7.6E-46
Pkinase_Tyr	P24941	7.8E-39
Pkinase_Tyr	O14757	3.6E-38
Pkinase_Tyr	Q16539	1.4E-33
Pkinase_Tyr	P11309	1.6E-24
PPARgamma_N	P37231	2.2E-35
Reprolysin_3	P39900	1.4E-06
RIO1	O60674	5.1E-06
SAM_1	Q9H2K2	1.5E-13
SAM_2	Q9H2K2	2.2E-16
SBP_bac_3	P39086	4.8E-21
SH2	P00519	1.5E-26
SH2	Q08881	1.9E-24
SH2	O60674	1.1E-09
SH3_1	Q08881	9.6E-18
SH3_1	P00519	2.3E-15
SH3_10	Q08881	1.5E-10
SH3_2	P00519	2.6E-10
SH3_2	Q08881	7.2E-09
SH3_3	P00519	8.8E-08
SH3_3	Q08881	3E-07
SH3_9	Q08881	1.6E-13
SH3_9	P00519	1.8E-11
SpoVT_C	Q9Y233	2.1E-08
TAD2	P04637	1.4E-20
TAXi_C	P56817	7.4E-14
TAXi_N	P56817	1.1E-09
Thrombin_light	P00734	7.9E-30
Trypsin	P03951	3.4E-76

Trypsin	P00742	6.9E-73
Trypsin	P00749	1.4E-72
Trypsin	P00734	7.2E-71
Trypsin_2	P03951	4E-09
Trypsin_2	P00742	3.6E-07
Vps39_1	Q9H2K2	5E-06
Y_phosphatase	P18031	8.3E-83
zf-C4	P03372	2.3E-34
zf-C4	P37231	2.8E-30
zf-C4	P10275	5.4E-30

Table S3. Pairwise binding pocket similarity scores for the 26 proteins in descending ordered by score. The similarity was calculated from the fingerprints generated using FuzCav. The table contains only similar pockets with a similarity score > 0.16 (as mentioned in Weill et al.2010)

Pocket A (PDB ID)	Pocket B (PDB ID)	Similarity Score
2c3i	4jia	0.19606
3up2	4jia	0.18789
4jia	4m0y	0.183051
4e5w	4jia	0.179241
2c3i	3up2	0.178001
2c3i	4m0y	0.17724
3pxf	4jia	0.175617
2c3i	4twp	0.173893
3up2	4e5w	0.171617
2y5h	3utu	0.170517
2c3i	3jvr	0.168414
3pxf	4m0y	0.16836
2pog	4m0y	0.164171
3up2	4m0y	0.163196
3jvr	4m0y	0.162516
2c3i	4dli	0.162483
4dli	4jia	0.161941
2c3i	3pxf	0.161829
3pxf	3udh	0.161829

Table S4. Optimal parameters for Random Forest models trained on six data splits using nested cross-validation. Data splits acronyms: BASR: Binding affinity-stratified random split, PS: Protein similarity-based split, BSS: Binding site similarity-based split, LS: Ligand similarity-based split, LWSR: Ligand weight-stratified random split, and LVSR: Ligand volume-stratified random split.

parameter	BASR	BSS	PS	LS	LWSR	LVSR
max_features	62	62	62	62	62	62
min_samples_leaf	1	1	1	1	1	1
min_samples_split	2	2	2	2	2	2
n_estimators	400	500	500	500	500	400

Table S5. Optimal parameters for Decision Tree models trained on six data splits using nested cross-validation. Data splits acronyms: BASR: Binding affinity-stratified random split, PS: Protein similarity-based split, BSS: Binding site similarity-based split, LS: Ligand similarity-based split, LWSR: Ligand weight-stratified random split, and LVSR: Ligand volume-stratified random split.

parameter	BASR	BSS	PS	LS	LWSR	LVSR
max_depth	None	None	None	None	None	None
max_features	62	auto	62	62	62	62
min_samples_leaf	10	10	10	10	10	10
min_samples_split	2	2	5	2	10	10

Table S6. Optimal parameters for Lasso Regression models trained on six data splits using nested cross-validation. Data splits acronyms: BASR: Binding affinity-stratified random split, PS: Protein similarity-based split, BSS: Binding site similarity-based split, LS: Ligand similarity-based split, LWSR: Ligand weight-stratified random split, and LVSR: Ligand volume-stratified split.

parameter	BASR	BSS	PS	LS	LWSR	LVSR
alpha	0.01	0.01	0.01	0.01	0.01	0.01

Table S7. Optimal parameters for Ridge Regression models trained on six data splits using nested cross-validation. Data splits acronyms: BASR: Binding affinity-stratified random split, PS: Protein similarity-based split, BSS: Binding site similarity-based split, LS: Ligand similarity-based split, LWSR: Ligand weight-stratified random split, and LVSR: Ligand volume-stratified split.

parameter	BASR	BSS	PS	LS	LWSR	LVSR
alpha	0.001	0.02	0.01	0.01	0.01	0.01

Additional file Figures

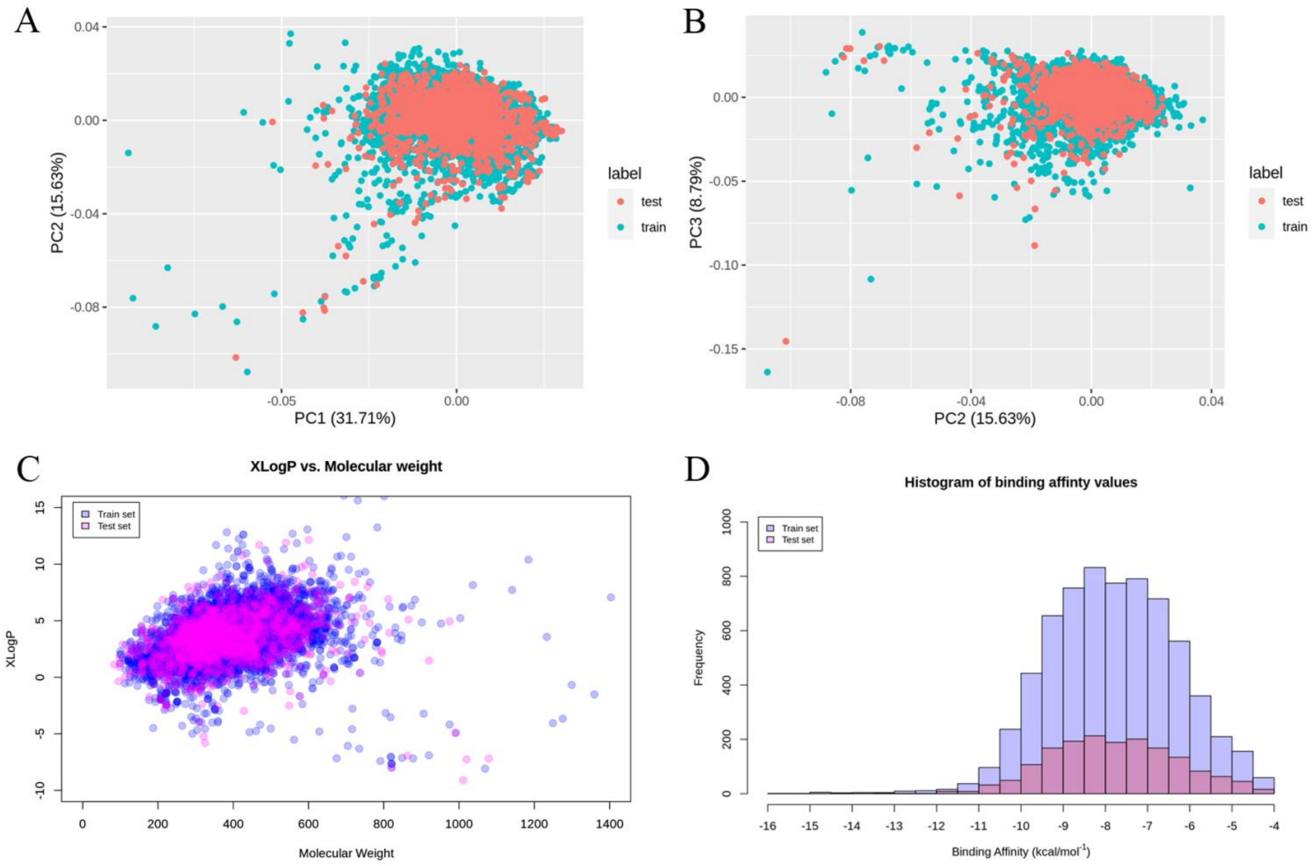

Figure S1. Diversity distribution of ligands in the random data split stratified on binding affinity. (A, B) Chemical space defined by PCA factorization; (C) chemical space defined by molecular weight as X-axis and XlogP as Y

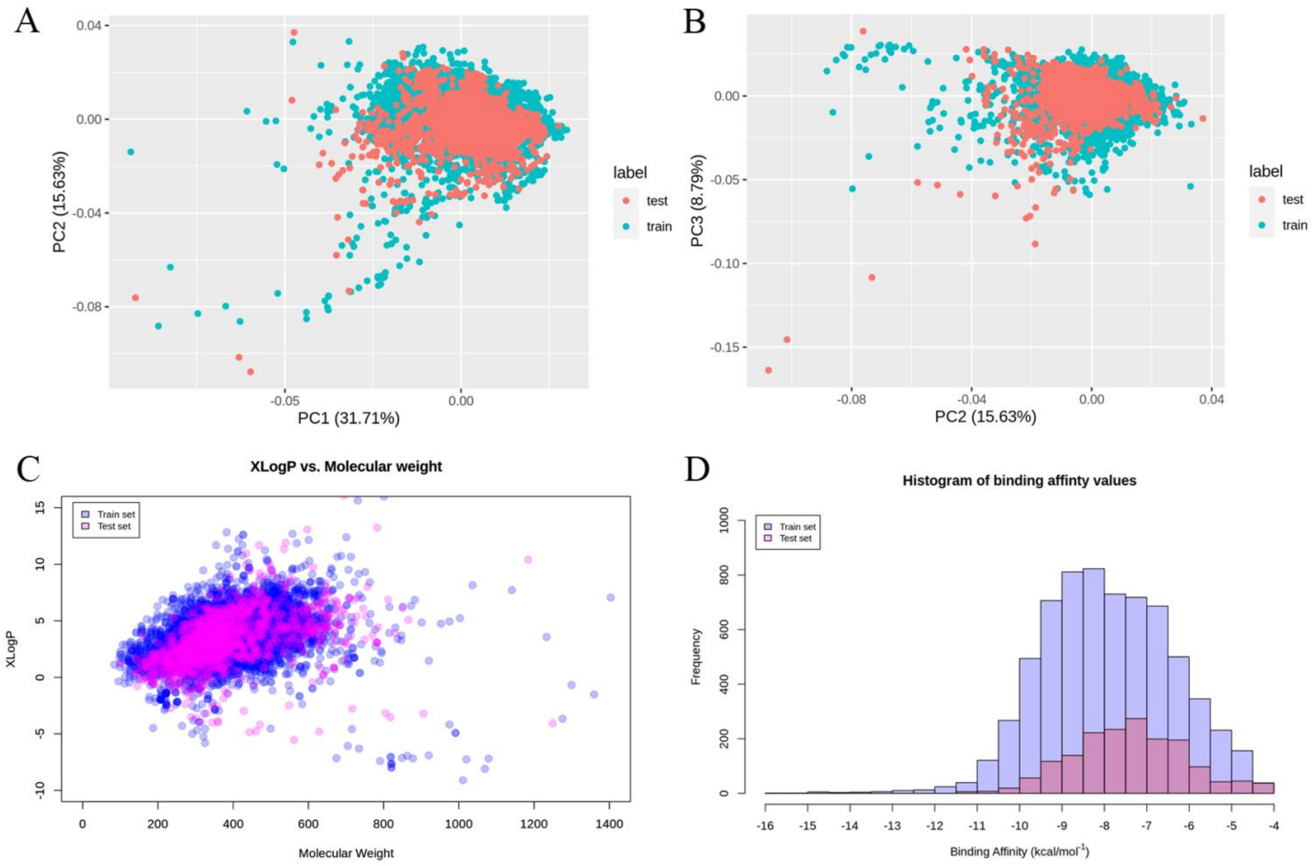

Figure 2 Diversity distribution of ligands in the protein similarity-based data split. (A, B) Chemical space defined by PCA factorization; (C) chemical space defined by molecular weight as X-axis and XlogP as Y

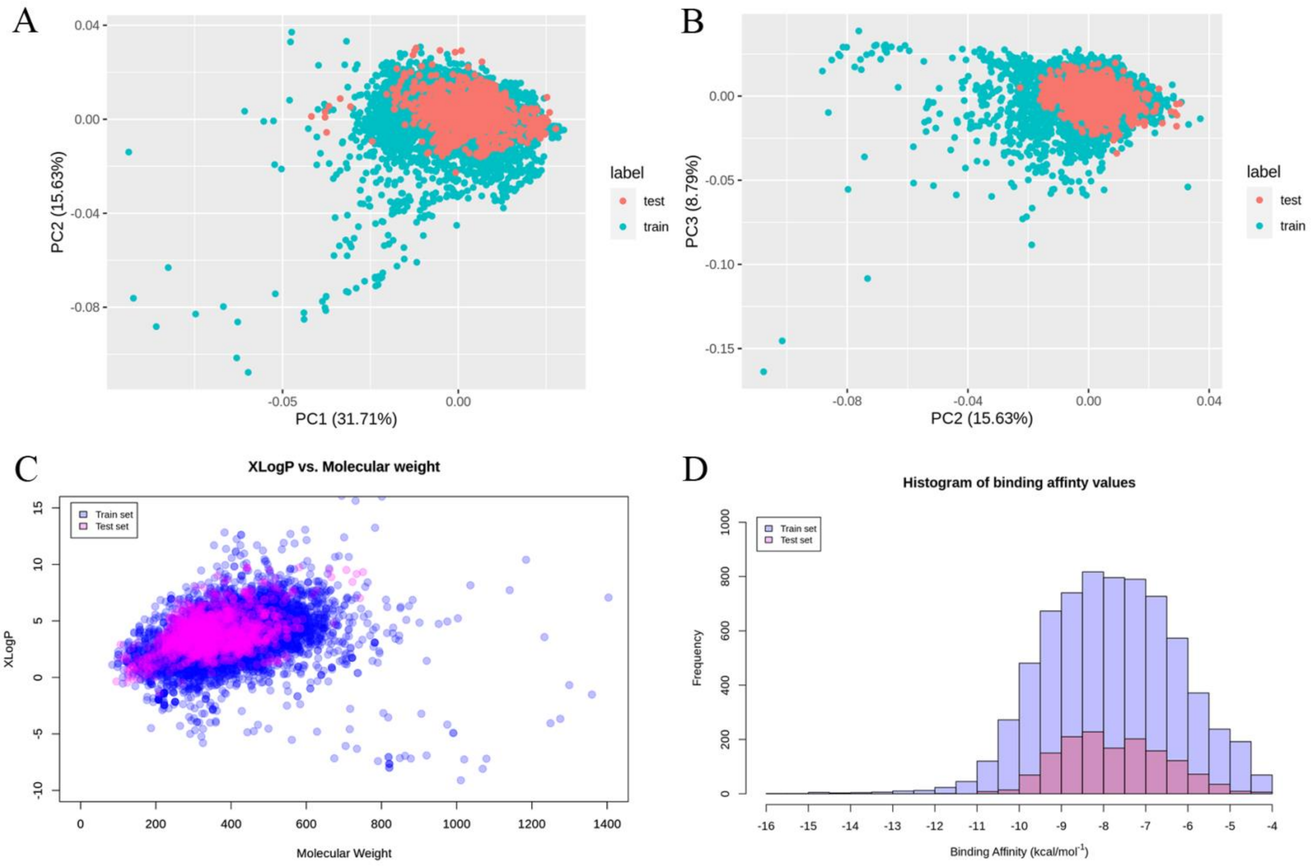

Figure 3 Diversity distribution of ligands in the binding pocket similarity-based data split. (A, B) Chemical space defined by PCA factorization; (C) chemical space defined by molecular weight as X-axis and XlogP as Y

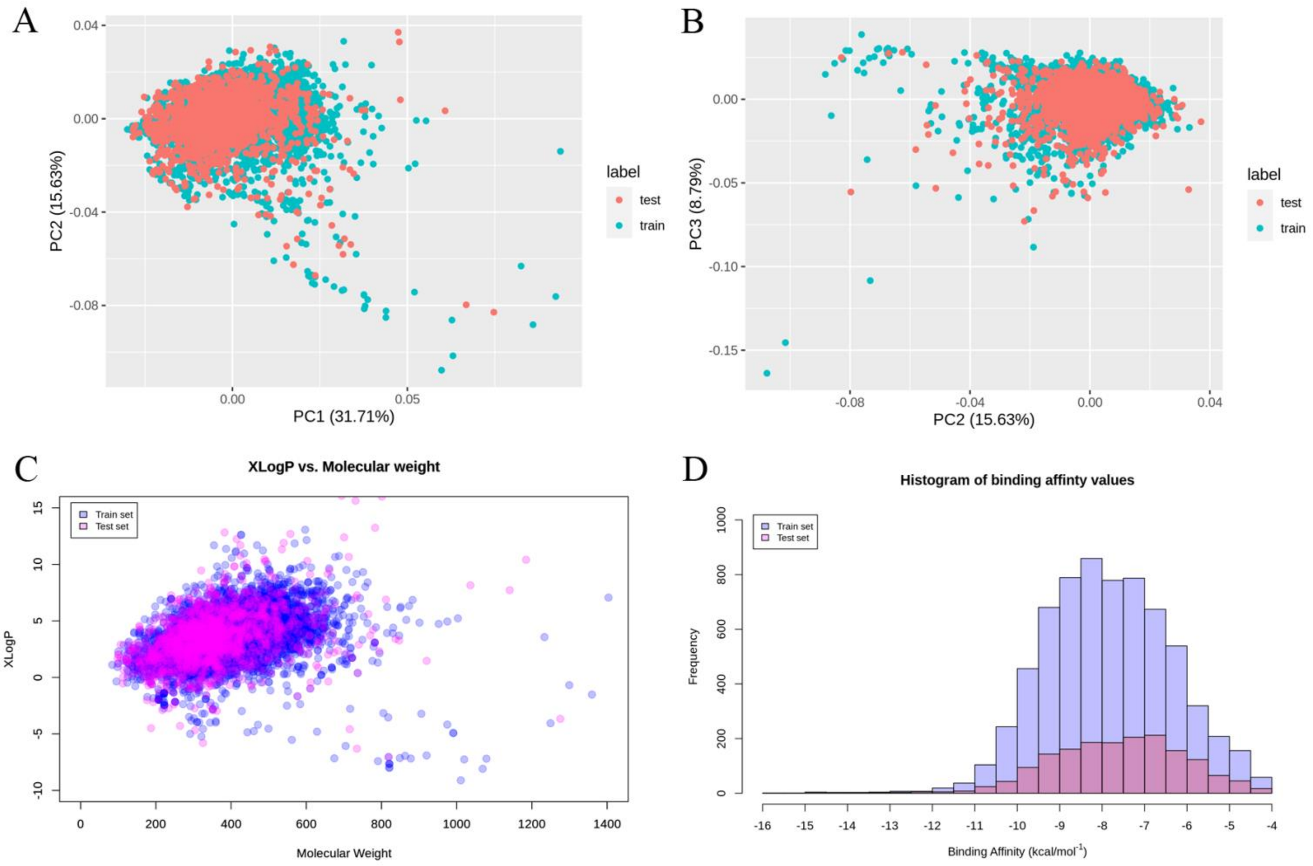

Figure 4 Diversity distribution of ligands in the ligand similarity-based data split. (A, B) Chemical space defined by PCA factorization; (C) chemical space defined by molecular weight as X-axis and XlogP as Y

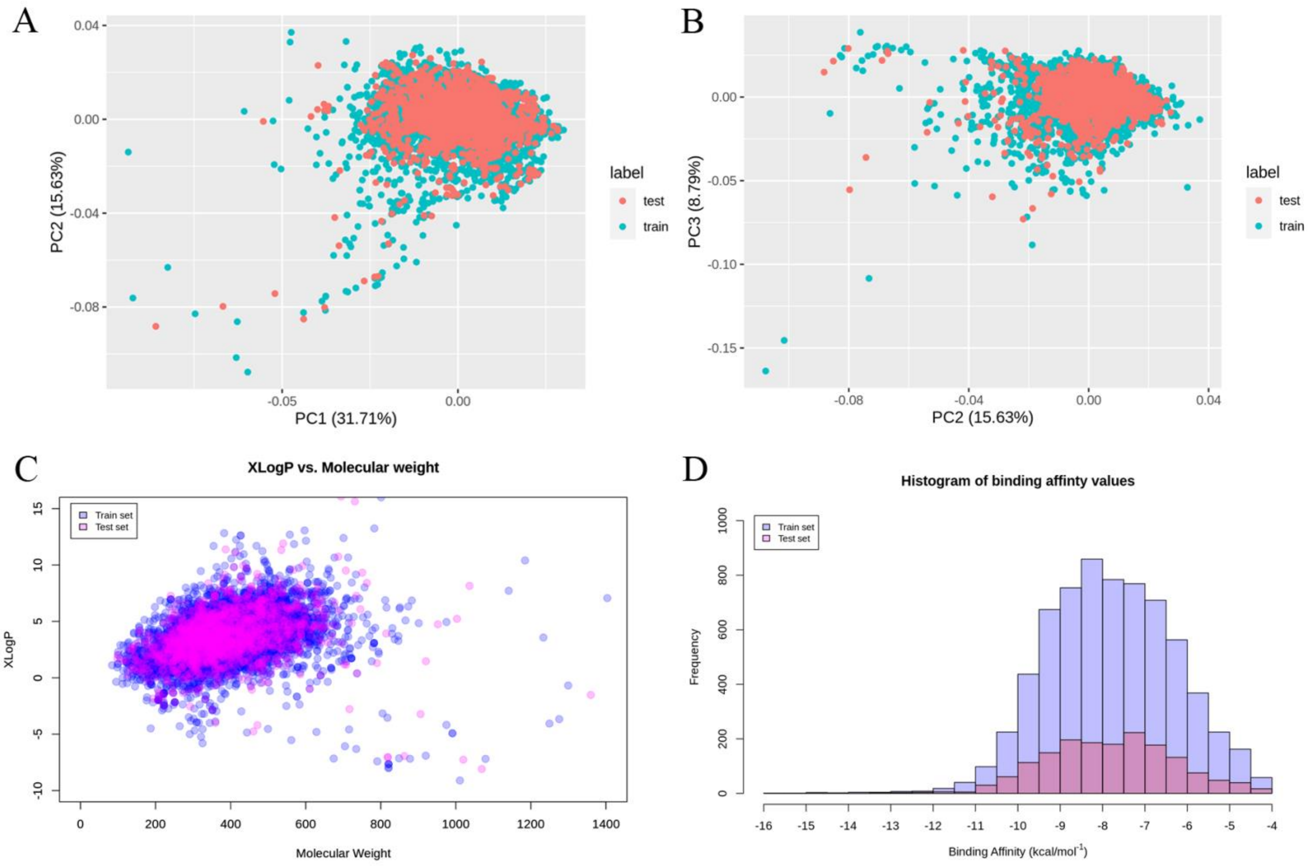

Figure 5 Diversity distribution of ligands in the in the random data split stratified on ligand volume. (A, B) Chemical space defined by PCA factorization; (C) chemical space defined by molecular weight as X-axis and XlogP as Y
